# Piperlongumine, a Potent Anticancer Phytotherapeutic, Induces Cell Cycle Arrest and Apoptosis In Vitro and In Vivo through the ROS/Akt Pathway in Human Thyroid Cancer Cells

**DOI:** 10.3390/cancers13174266

**Published:** 2021-08-24

**Authors:** Fang-Ping Kung, Yun-Ping Lim, Wen-Ying Chao, Yi-Sheng Zhang, Hui-I Yu, Tsai-Sung Tai, Chieh-Hsiang Lu, Shu-Hsin Chen, Yi-Zhen Li, Pei-Wen Zhao, Yu-Pei Yen, Ying-Ray Lee

**Affiliations:** 1 Division of Endocrinology and Metabolism, Department of Internal Medicine, Ditmanson Medical Foundation Chia-Yi Christian Hospital, Chiayi 60002, Taiwan; 07266@cych.org.tw (F.-P.K.); 04490@cych.org.tw (H.-I.Y.); 04015@cych.org.tw (T.-S.T.); 02602@cych.org.tw (C.-H.L.); 15159@cych.org.tw (Y.-P.Y.); 2 Department of Pharmacy, College of Pharmacy, China Medical University, Taichung 406040, Taiwan; limyp@mail.cmu.edu.tw; 3 Department of Internal Medicine, China Medical University Hospital, Taichung 404332, Taiwan; 4 Department of Medical Research, China Medical University Hospital, Taichung 404332, Taiwan; 5 Department of Nursing, Min-Hwei College of Health Care Management, Tainan 73658, Taiwan; A129@o365.mhchcm.edu.tw; 6 Department of Medical Research, Ditmanson Medical Foundation Chia-Yi Christian Hospital, Chiayi 60002, Taiwan; s1030722@alumni.ncyu.edu.tw (Y.-S.Z.); 10472@cych.org.tw (S.-H.C.); 10862@cych.org.tw (Y.-Z.L.); 14421@cych.org.tw (P.-W.Z.); 7 Department of Microbiology and Immunology, School of Medicine, College of Medicine, Kaohsiung Medical University, Kaohsiung 80708, Taiwan

**Keywords:** novel therapeutic strategy, safe anticancer treatment, anaplastic thyroid cancer, recurrent thyroid cancer, effective treatment

## Abstract

**Simple Summary:**

There is no effective treatment currently available for patients with anaplastic, recurrent papillary, or follicular thyroid cancers. Reactive oxygen species (ROS) are believed to hold promise as a new therapeutic strategy for multiple human cancers. However, studies on ROS inducers for human thyroid cancer treatment are scarce. This study assesses the anticancer activity and the detailed downstream mechanisms of piperlongumine, a ROS inducer, in human thyroid cancer cells. We demonstrate that piperlongumine inhibits cell proliferation, regulates the cell cycle, and induces cellular apoptosis in various types of human thyroid cancer cells. The antihuman thyroid cancer activity of piperlongumine was through ROS induction, and it further suppressed the downstream Akt signaling pathway to elevate mitochondria-dependent apoptosis. A mouse xenograft study demonstrated that piperlongumine was safe and could inhibit tumorigenesis in vivo. The present study provides strong evidence that piperlongumine can be used as a therapeutic candidate for human thyroid cancers.

**Abstract:**

Thyroid cancer (TC) is the most common endocrine malignancy, and its global incidence has steadily increased over the past 15 years. TC is broadly divided into well-differentiated, poorly differentiated, and undifferentiated types, depending on the histological and clinical parameters. Thus far, there are no effective treatments for undifferentiated thyroid cancers or advanced and recurrent cancer. Therefore, the development of an effective therapeutic is urgently needed for such patients. Piperlongumine (PL) is a naturally occurring small molecule derived from long pepper; it is selectively toxic to cancer cells by generating reactive oxygen species (ROS). In this study, we demonstrate the potential anticancer activity of PL in four TC cell lines. For this purpose, we cultured TC cell lines and analyzed the following parameters: Cell viability, colony formation, cell cycle, apoptosis, and cellular ROS induction. PL modulated the cell cycle, induced apoptosis, and suppressed tumorigenesis in TC cell lines in a dose- and time-dependent manner through ROS induction. Meanwhile, an intrinsic caspase-dependent apoptosis pathway was observed in the TC cells under PL treatment. The activation of Erk and the suppression of the Akt/mTOR pathways through ROS induction were seen in cells treated with PL. PL-mediated apoptosis in TC cells was through the ROS-Akt pathway. Finally, the anticancer effect and safety of PL were also demonstrated in vivo. Our findings indicate that PL exhibits antitumor activity and has the potential for use as a chemotherapeutic agent against TC. This is the first study to show the sensitivity of TC cell lines to PL.

## 1. Introduction

Thyroid cancer (TC) is the most common malignancy of the endocrine organs, and its incidence has steadily increased in the past 15 years [1,2]. Most tumors (>95% of TCs) are derived from follicular cells, whereas, some tumors (medullary TC) are derived from C cells. Follicular cell-derived TCs are broadly divided into well-differentiated (DTC), poorly differentiated (PDTC), and anaplastic (ATC) types, depending on the histological and clinical parameters. DTC includes papillary TC (PTC) and follicular TC (FTC); most DTCs have a slowly progressive course and a normally favorable prognosis. Although the initiated treatment in most patients with PTC and FTC is effective, 10–15% of patients have tumor recurrence. By contrast, ATC is rare; this type is highly aggressive and 100% lethal within one year after diagnosis [3,4]. Currently, there is no effective treatment for ATC, because the patients fail to respond to the available radiotherapy and chemotherapeutic agents [5]. Therefore, the development of an effective therapeutic for ATC and recurrent DTC and PDTC is urgently needed.

Piperlongumine (PL), also known as piplartine, is an amide alkaloid isolated from long pepper, *Piper longum* L. [6]. PL exerts extensive biological activities, including antiplatelet, antimicrobial, antiangiogenetic, antidiabetic, antidepressant, antiatherosclerotic, neuroprotective, and anticancer properties [7]. PL also exerts antitumor activity in selectively affected transformed cell types in lymphoma [8], melanoma [9], glioblastoma [10], oral [11], head and neck [12], lung [13], breast [14], liver [15], cholangiocarcinoma [16], renal [17], pancreatic [18], gastric [19], colon [20], bladder [21], and prostate [22] cancers, without affecting normal cells. The anticancer activities occur through the p38/JNK, MAPK, and NF-κB pathways and by the induction of high levels of reactive oxygen species (ROS) [16,23,24]. PL also causes cell death through both caspase-dependent apoptosis and necrosis and induces the downregulation of Bcl2 expression and the activation of caspase-3, poly (ADP-ribose) polymerase (PARP), and JNK [16,24]. PL has shown antitumor activity in several whole-animal models, and it is highly safe when used in vivo [25,26]. However, no study has provided evidence for the effects of PL against TC. In the present study, we aimed to evaluate the therapeutic effects of PL in human multidrug-resistant PTC, FTC, and ATC cells. Furthermore, the anticancer mechanisms of PL were also determined in these cells, and a mouse xenograft model was used to confirm the therapeutic property of PL in vivo.

## 2. Results

### 2.1. Piperlongumine Inhibits Cell Proliferation and Colony Formation of Human Thyroid Cancer Cells

**Figure 1:**
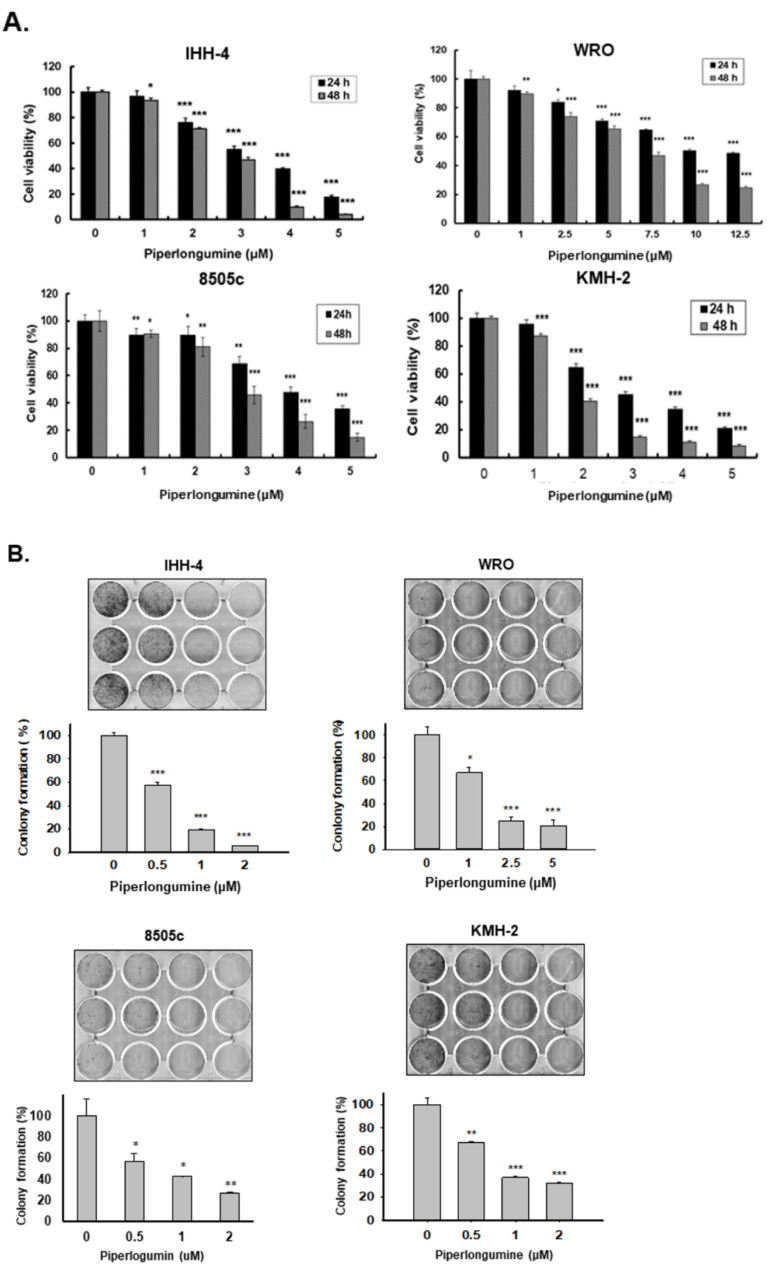
Suppression of tumor growth and colony formation by piperlongumine (PL) in vitro in human thyroid cancer cells. Three types of human thyroid cancer cells, namely, papillary thyroid cancer (PTC) (IHH-4), follicular thyroid cancer (FTC) (WRO), and anaplastic thyroid cancer (ATC) (8505c and KMH-2), were incubated with PL, and the (**A**) cellular viability and (**B**) colony formation were examined. Dimethyl sulfoxide (DMSO) was used as a negative control. Three independent experiments of cellular viability were conducted. Compared with the control group, * indicates *p* < 0.05, ** indicates *p* < 0.01, *** indicates *p* < 0.001.

To investigate the effects of PL in human TCs, we performed the cell viability test using the CCK-8 assay on four human TC cell lines (IHH-4, WRO, 8505c, and KMH-2; see Section 4.1) treated with various concentrations of PL (0, 1, 2, 3, 4, and 5 µM) for 24 and 48 h. Cell growth was inhibited by PL treatment in each cell line in a dose- and time-dependent manner (Figure 1A), suggesting that PL could suppress cell growth in various types of human TC cells. The IC_50_ values of PL in these cells are shown in Table 1. Among them, KMH-2 was more sensitive to PL treatment, and distinctly, WRO was more resistant when incubated with PL. In addition, to assess whether PL could suppress cellular proliferation, an in vitro colony formation assay was conducted, and the results showed that PL could significantly reduce colony formation in a dosage-dependent manner (Figure 1B). Moreover, IHH-4 cells were more sensitive than the other cell lines in the PL anticolony formation analysis (Figure 1B). Altogether, we demonstrated that PL is a potential anticancer agent against multiple human TCs. Further, PL doses of 2 and 3 μM were selected as effective doses for subsequent experiments owing to their abilities to suppress IHH-4, 8505c, and KMH-2 cells below 50% of the control.

### 2.2. PL Induces G2/M Phase Cell Cycle Arrest and Cellular Apoptosis through the Intrinsic Caspase-Dependent Pathway in Human Thyroid Cancer Cells

**Figure 2:**
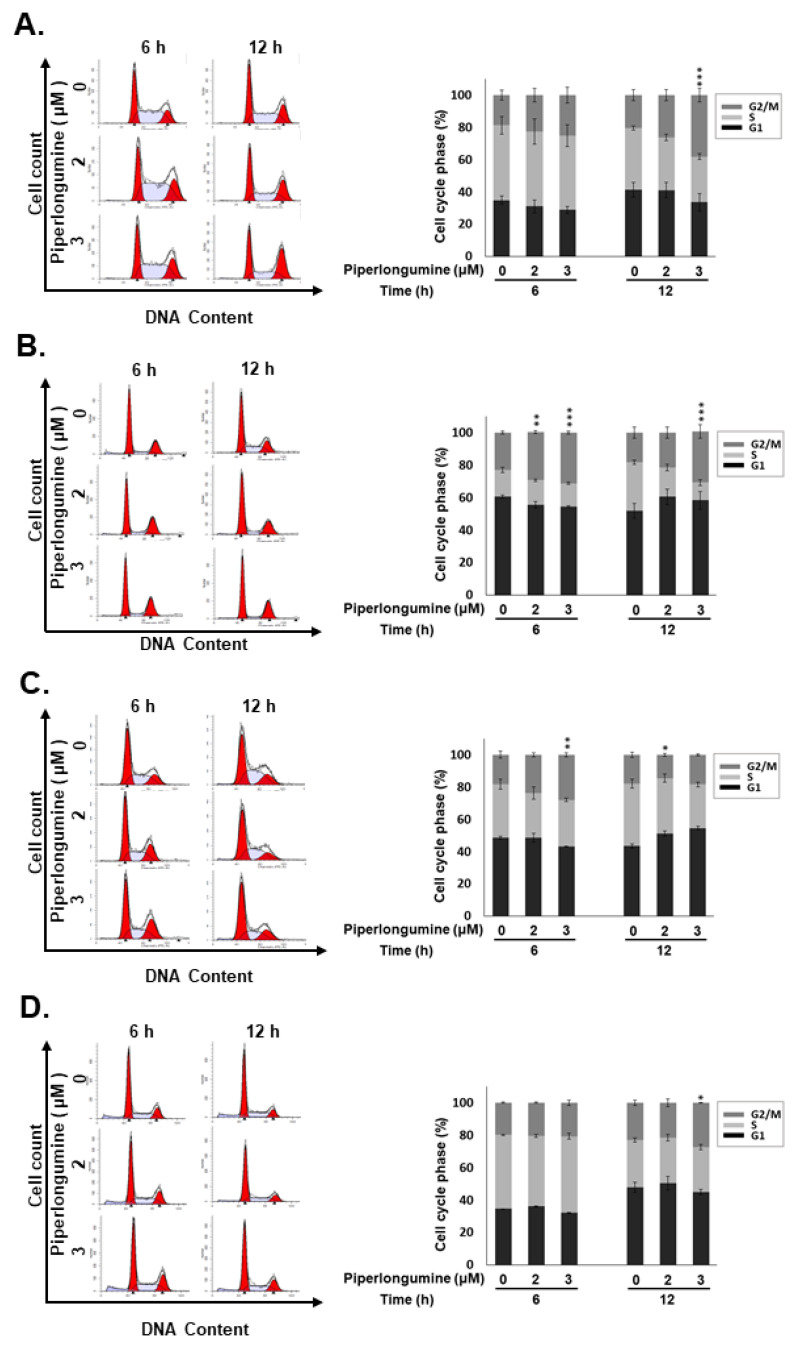
Induction of cell cycle arrest at the G2/M phase by piperlongumine (PL) in human thyroid cancer cells. Human thyroid cancer cells, namely, (**A**) IHH-4, (**B**) WRO, (**C**) 8505c, and (**D**) KMH-2 cells, were incubated with a control medium or PL, and the cell cycle was examined with fluorescence-activated cell sorting (FACS) flow cytometry. Dimethyl sulfoxide (DMSO) was used as a negative control. Three independent experiments were conducted. * indicated comparing with the untreated group. * indicates *p* < 0.05. ** indicates *p* < 0.01. *** indicates *p* < 0.001.

**Figure 3:**
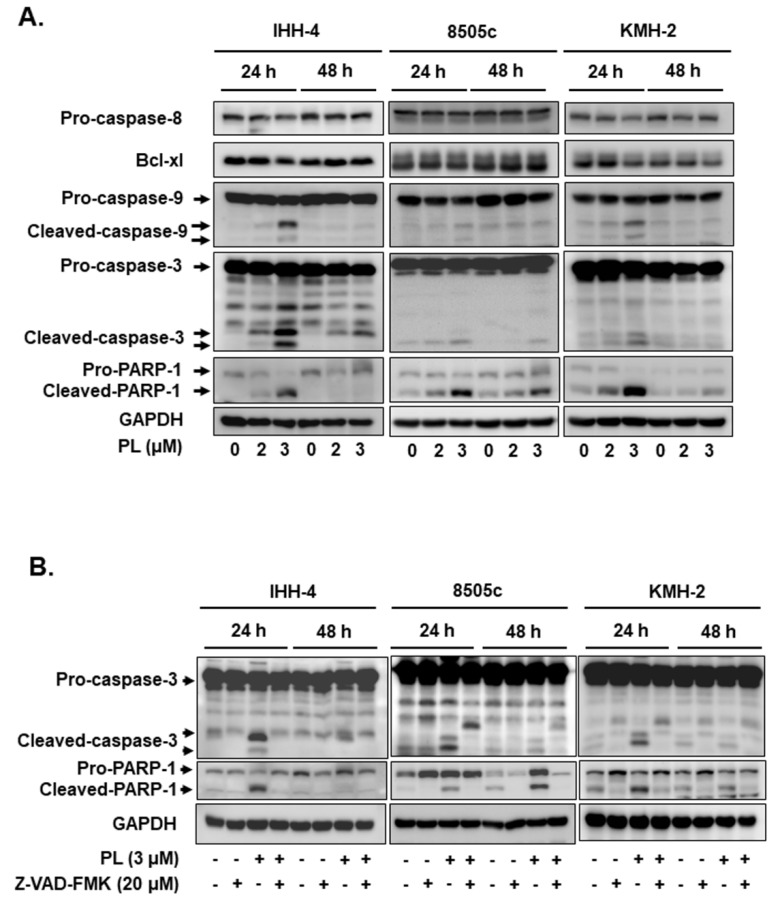

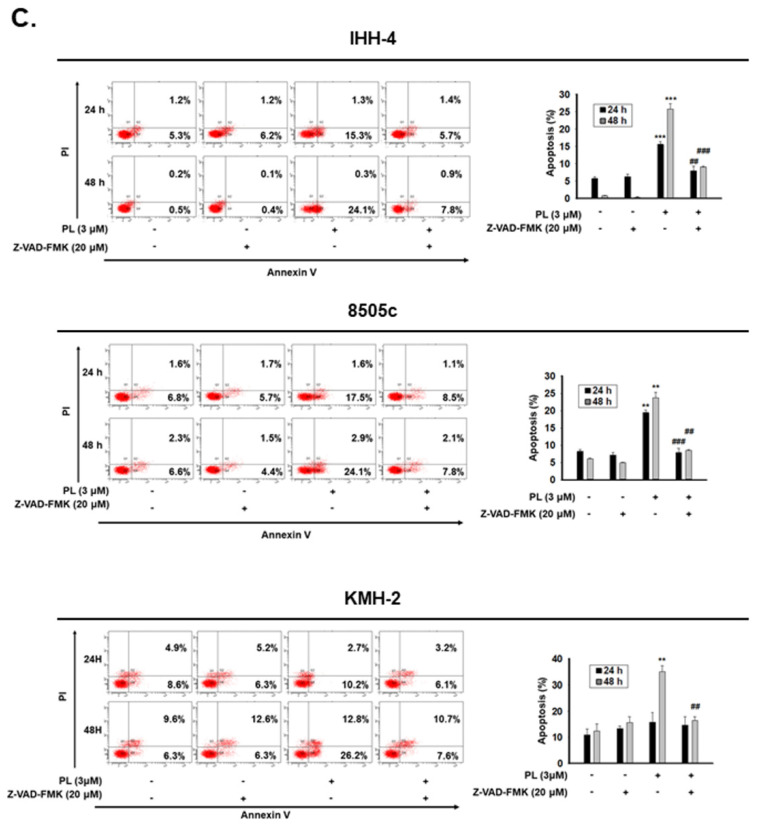
Activation of mitochondria-dependent cellular apoptosis by piperlongumine (PL) in human thyroid cancer cells. (**A**) Three human thyroid cancer cells were incubated with PL, and the expression of caspases and poly (ADP-ribose) polymerase (PARP) was examined by western blotting. (**B**) Z-VAD-FMK was used to suppress PL-mediated caspase-dependent apoptosis, and the activation of caspase and PARP was assessed by western blots, and (**C**) the cellular apoptosis was determined with flow cytometry. Dimethyl sulfoxide (DMSO) was used to be a negative control. GAPDH was used as a loading control. Three independent experiments were conducted. The data include western blotting, and the flow cytometry dot plot was shown with a representative experiment. * indicated comparing with the untreated group and # showed comparing with PL treated group. ** and ## indicates *p* < 0.01. *** and ### indicates *p* < 0.001.

Because PL treatment reduced the viability of TC cells (Figure 1), we further examined whether growth inhibition by PL led to cell cycle alteration and apoptosis. Cell cycle analysis indicated that a large cell population was in the G2/M phase with 6–12 h treatment in all the TC cell lines after PL treatment (Figure 2), suggesting that PL could promote cell cycle arrest at the G2/M phase in human TCs. Cellular apoptosis was also observed to occur in a dosage-dependent manner in all the cells treated with PL (Figure 3A). Moreover, the expression or the activation of caspases, such as caspase-8, caspase-9, and caspase-3, and PARP were examined. As shown in Figure 3A, caspase-9, caspase-3, and PARP were activated due to PL treatment in a dose-dependent manner. However, caspase-8 was not activated in PL-mediated apoptosis. These data suggest that PL induced cellular apoptosis in KMH-2 and IHH-4 cells through an intrinsic pathway, and the extrinsic pathway was not involved.

To unravel whether caspase activation is involved in PL-mediated cellular apoptosis in IHH-4, 8505c, and KMH-2 cells, the Z-VAD-FMK was used. The activation of caspase-3 and PARP were significantly reversed in the cells with pre-treatment of Z-VAD-FMK (Figure 3B), confirming that PL can induce a caspase activation in human TC cells. Furthermore, PL-induced cellular apoptosis was significantly reversed in the cells pre-incubated with Z-VAD-FMK (Figure 3C), demonstrating that caspase-dependent apoptosis occurred in IHH-4, 8505c, and KMH-2 cells when treated with PL. Altogether, our findings demonstrate that PL inhibited cell growth and induced apoptosis in human TC cell lines through an intrinsic caspase-dependent pathway.

### 2.3. ROS Induction Is Involved in PL-Mediated Antitumor Behavior in Human Thyroid Cancer Cells

**Figure 4:**
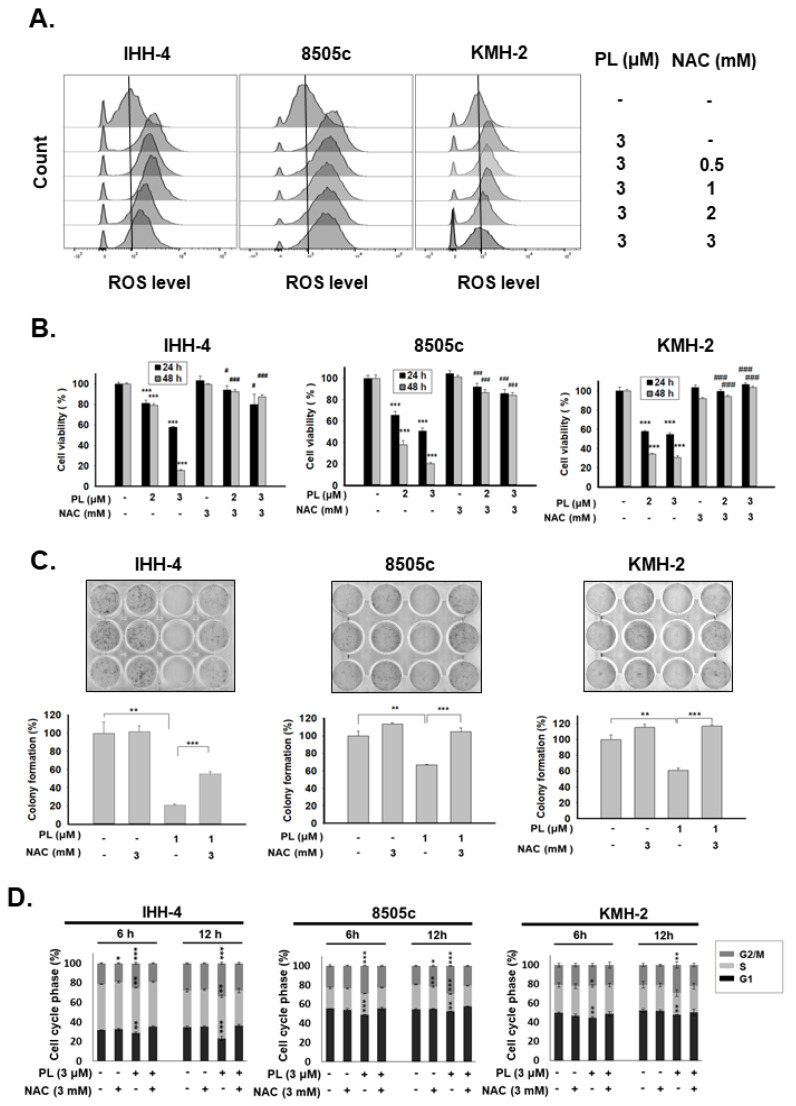
Modulation of cell growth, tumorigenesis, and cell cycle by piperlongumine (PL) by inducing reactive oxygen species (ROS) in human thyroid cancer cells. Three human thyroid cancer cells were incubated with PL, and NAC was used to inhibit ROS activation. (**A**) The expression of ROS was determined with fluorescence-activated cell sorting (FACS) flow cytometry. (**B**) Cellular viability and (**C**) colony formation were examined. (**D**) Cell cycle regulation was determined by FACS flow cytometry. Dimethyl sulfoxide (DMSO) was used as a negative control. ROS expression in (**A**) was confirmed with two independent experiments, and a representative experiment was shown. The cell viability, colony formation, and cell cycle were obtained in three independent experiments. * indicated comparing with the untreated group and # showed comparing with PL treated group. * and # indicates *p* < 0.05. ** indicates *p* < 0.01. *** and ### indicates *p* < 0.001.

PL exerts anticancer activity in various types of cancers by promoting the induction of ROS [27]. In the present study, the induction of ROS in IHH-4, 8505c, and KMH-2 cells under PL treatment was also determined (Figure 4A). Pre-treatment with N-acetylcysteine (NAC; a selective ROS scavenger) significantly reduced PL-mediated ROS activation (Figure 4A). Moreover, the suppression of PL-induced ROS using NAC showed significant reversal of cytotoxic effects of PL in IHH-4, 8505c, and KMH-2 cells, according to cellular viability and colony formation (Figure 4B,C). In addition, cells pre-treated with NAC showed complete reversal of PL-induced cell cycle progression at the G2/M phase (Figure 4D).

**Figure 5:**
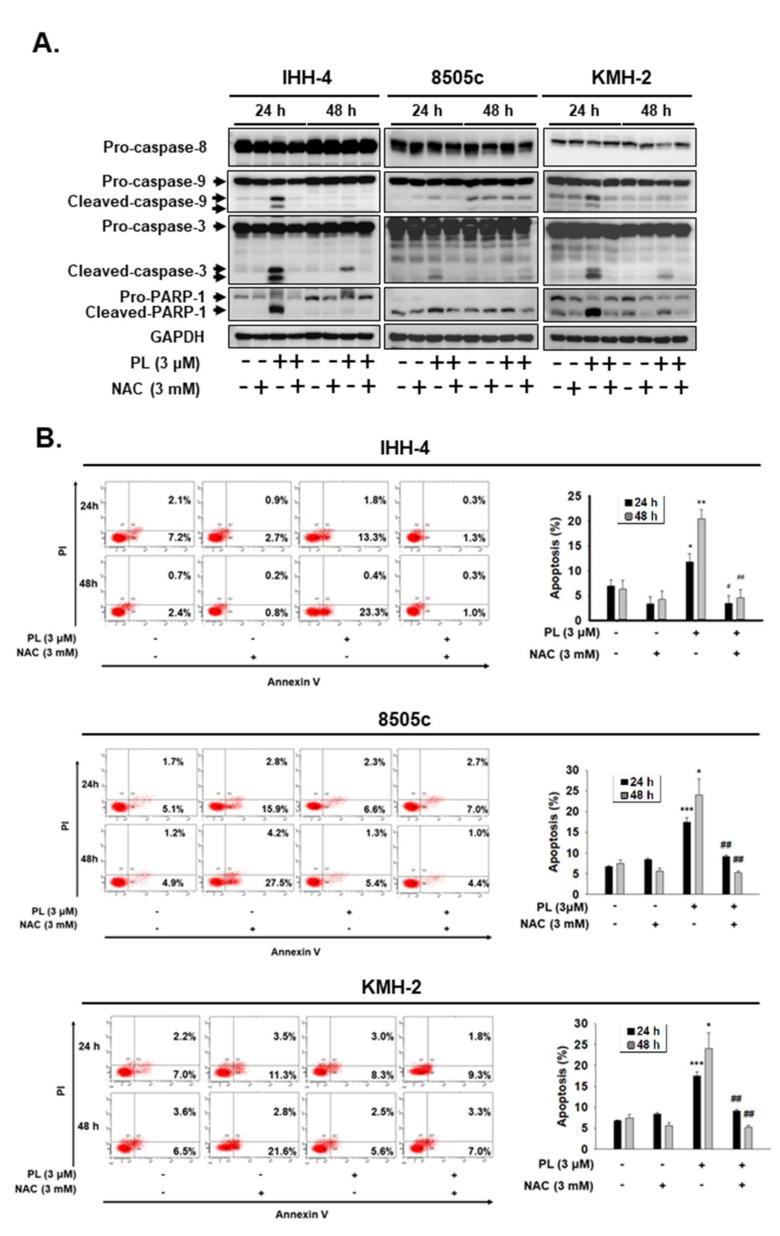
Piperlongumine (PL)-mediated cellular apoptosis was regulated by inducing reactive oxygen species (ROS) in human thyroid cancer cells. Three human thyroid cancer cells were treated with PL; (**A**) the activation of caspase and poly (ADP-ribose) polymerase (PARP) and (**B**) cellular apoptosis were investigated by western blotting and fluorescence-activated cell sorting (FACS) flow cytometry. NAC was used to suppress PL-induced ROS. DMSO was used as a negative control. GAPDH was used as a loading control. Three independent experiments each for western blotting and flow cytometry were conducted, and western blots and the flow cytometry dot plots of a representative experiment are shown. * indicated comparing with the untreated group and # showed comparing with PL treated group. * and # indicates *p* < 0.05. ** and ## indicates *p* < 0.01. *** indicates *p* < 0.001.

Furthermore, the role of ROS in PL-mediated cellular apoptosis in IHH-4, 8505c, and KMH-2 cells was also evaluated. The activation of caspase-9, caspase-3, and PARP were determined in these cells under PL treatment; however, the activation of caspases and PARP in these cells after incubation with PL was reduced in the presence of NAC (Figure 5A). Moreover, the number of apoptotic cells significantly increased under PL treatment, but these effects were blocked by pre-treatment with NAC (Figure 5B). Taken together, these findings indicate that PL induces TC apoptosis and cell cycle arrest, as well as in vitro tumorigenesis, by generating and inducing intracellular ROS.

### 2.4. PL Induces Cellular Apoptosis through the ROS-Modulated Akt Pathway

**Figure 6:**
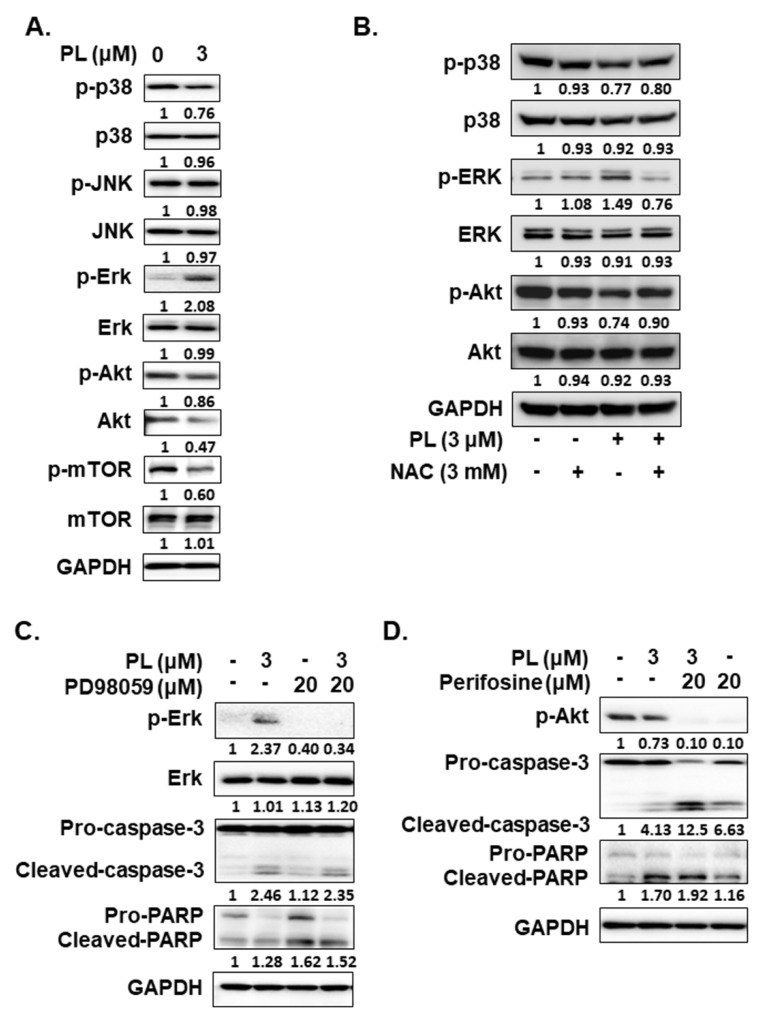
Regulation of the activation of the Erk and Akt signaling pathways by piperlongumine (PL) through reactive oxygen species (ROS) induction. KMH-2 cells were used to evaluate the regulation of signaling pathways under PL treatment. (**A**) The expression and the activation of Erk, JNK, p38, Akt, and mTOR were examined by western blotting in PL-treated cells. (**B**) NAC was used to block PL-mediated ROS induction and p38 activation, and the resulting expression of Erk and Akt was examined by western blotting. (**C**) PD98059 was used to suppress PL-mediated Erk activation, and (**D**) perifosine was used to promote PL-mediated Akt activation, and the activation of caspase-3 and PARP was examined by western blotting. DMSO was used as a negative control. GAPDH was used as a loading control. Three independent experiments were conducted, and a representative experiment is shown.

**Figure 7:**
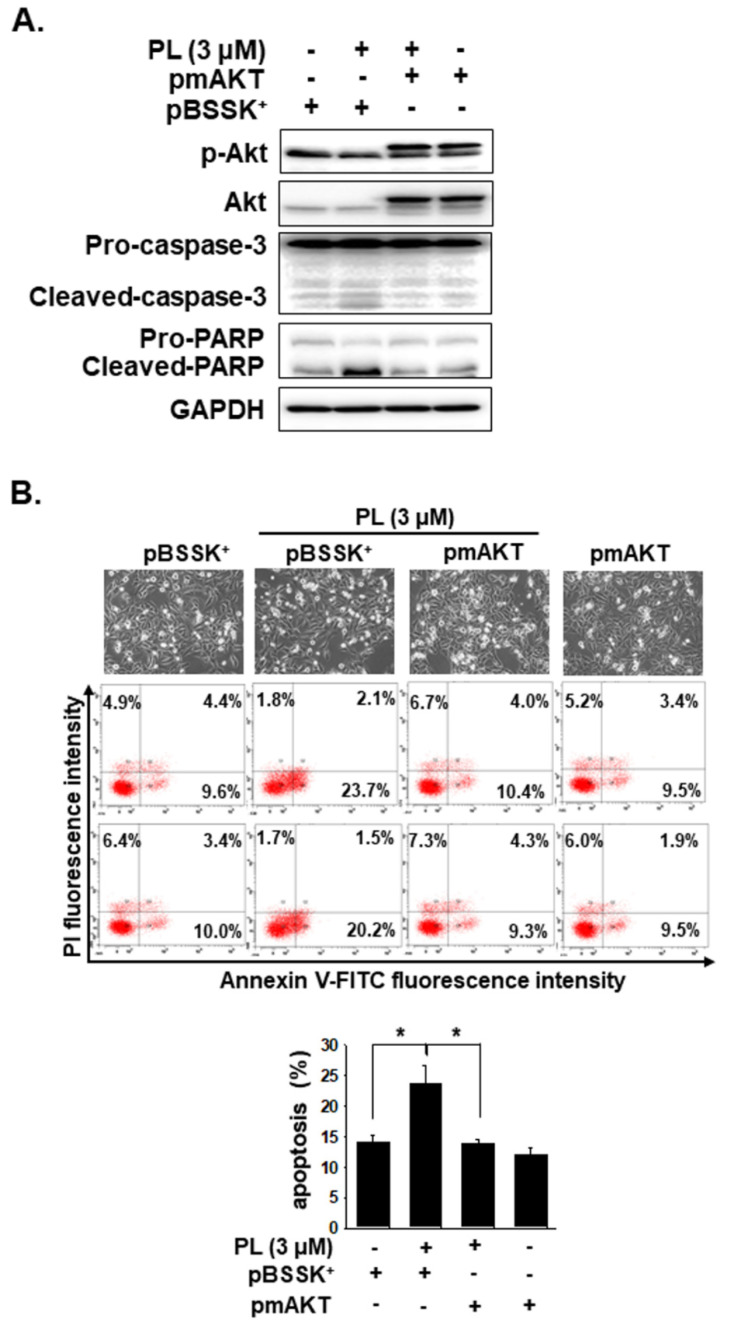
Piperlongumine (PL) induced cellular apoptosis through the Akt signaling pathway. KMH-2 cells were used to verify the involvement of the Akt signaling pathway in PL-caused apoptosis. (**A**) KMH-2 cells were transfected with or without constitutively active Akt construct, and the expression and the activation of Akt, caspase-3, and PARP were investigated by western blots after incubation with PL for 24 h. (**B**) Cellular apoptosis was investigated by flow cytometry. pBSSK^+^ was used as a negative control of transfection. pmAKT was a constitutively active form of the ATK construct. DMSO was used as a negative control. GAPDH was used as a loading control. Three independent experiments each for western blotting and flow cytometry were conducted, and the western blots and flow cytometry dot plots of a representative experiment are shown. * indicates *p* < 0.05.

ROS is involved in apoptosis regulation by regulating Erk, JNKs, p38, and through the Akt/mTOR pathways in many types of cancers [28,29]. Thus, we further assessed whether Erk, JNK, p38, and Akt activation are involved in regulating the apoptotic effect caused by PL. PL increased p-ERK expression, but decreased the expression of p-p38, p-Akt, and p-mTOR in KMH-2 cells (Figure 6A). Because ROS activation was found to occur in these cells under PL treatment (Figure 4), herein, the relationship of ROS with Erk, p38, and Akt pathways was further examined. The activation of Erk and the suppression of the Akt pathway under PL treatment were found to be reversed by NAC pre-treatment (Figure 6B). However, p38 activation in PL-treated cells showed no significant difference without or with co-incubation with NAC (Figure 6B). This finding suggested that PL-mediated ROS activation may be the upstream pathway of the Erk and Akt pathways. PL-mediated ROS activation could induce TC apoptosis and regulate the activation of the Erk and Akt pathways. We further evaluated the activation of caspase-3 and PARP in cells treated with PL in combination with or without PD98059 (an inhibitor of Erk) and perifosine (an inhibitor of Akt). We demonstrated that reducing PL-mediated Erk activation could not reverse PL-mediated cellular apoptosis (Figure 6C), and an increase in PL-mediated Akt inactivation could promote PL-mediated cellular apoptosis (Figure 6D). In addition, a dominant active Akt construct was transfected to reverse PL-suppressed Akt activation in KMH-2 cells, and the PL-mediated activation of caspase-3 and PARP was found to be inhibited (Figure 7A). Moreover, the PL-mediated cellular apoptosis was significantly reduced in KMH-2 cells in the presence of constitutive Akt activation (Figure 7B). These findings demonstrate that PL treatment in TC cells can activate ROS, reduce Akt signaling, and finally, induce cellular apoptosis.

### 2.5. PL Reduces Tumor Growth and Induces Tumor Cell Apoptosis In Vivo

**Figure 8:**
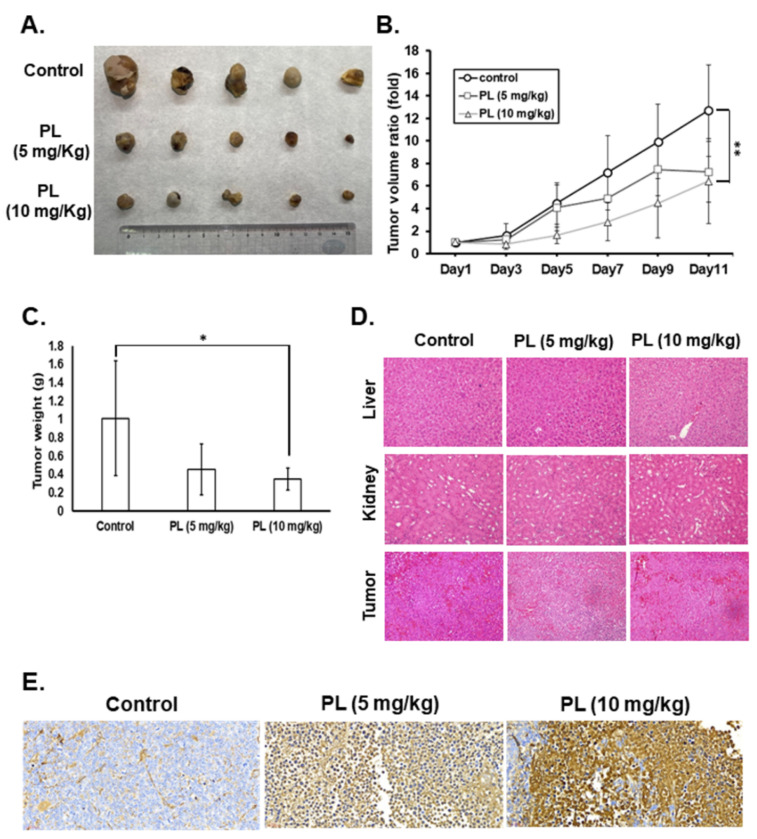
Piperlongumine significantly inhibited human thyroid cancer cell growth in vivo. IHH-4-bearing mice were treated with either dimethyl sulfoxide (DMSO) (*n* = 5) or PL (*n* = 5/group). (**A**) Representative images of excised tumors from each group. (**B**) Tumor volume and (**C**) tumor weight were measured to reflect tumor growth in vivo. (**D**) Hematoxylin and eosin staining to determine cellular pathology and immune cell infiltration. 200× magnification. (**E**) Apoptosis in tumors was measured by terminal deoxynucleotidyl transferase-mediated nick end labeling (TUNEL) staining, and the brown staining indicates apoptotic cells. 40× magnification. DMSO was used as a negative control. * indicates *p* < 0.05. ** indicates *p* < 0.01.

PL displays a good antitumor behavior in various human cancers [23]. We demonstrated that PL exerts anticancer activities, such as cell cycle arrest, growth inhibition, and apoptosis induction, and suppresses colony formation in vitro. In the present study, a mouse xenograft model was used to evaluate the anti-TC activity and the safety of PL in vivo. Nude mice received a subcutaneous injection of IHH-4 cells into the right flank and intraperitoneal injection of PL at various doses. Tumorigenesis was examined every two days, and the bodyweight of mice was also recorded. The tumor volumes in the mice treated with PL (10 mg/kg) were significantly lower than those in the control groups (Figure 8A,B). Moreover, the tumor weight in the PL (10 mg/kg) treatment group significantly decreased when compared with that in the control group (Figure 8C). To address the safety of PL in this study, we assessed the bodyweight and the pathological phenomena of mouse tissues by (hematoxylin and eosin) HE staining. The bodyweight of mice showed no significant difference between the control and PL treatment groups. Furthermore, no pathological findings in the liver and kidney were detected in the control or PL treatment groups (Figure 8D). In addition, there was no significant infiltration of immune cells in any of the tissues (Figure 8D). Finally, tumor cell apoptosis in vivo was assessed with the TUNEL assay, which revealed that PL treatment could induce tumor apoptosis in a dose-dependent manner in vivo (Figure 8E). These data demonstrate that PL exhibits good therapeutic action against human TCs and is safe for treatment in vivo.

## 3. Discussion

TC predominantly affects women worldwide; among all TCs, ATC and recurrent PTC and FTC display a highly aggressive nature. Currently, no effective treatments have been reported. Recently, it is growing interest to develop therapeutic compounds from natural products. Our findings indicate that PL induces a ROS-mediated mechanism, which perturbs numerous cellular signaling pathways and finally elevates apoptosis in TC cells. This finding is based on two crucial findings. Primary, ROS accumulation happened in PL-incubated cells. Second, blocking of ROS with NAC considerably suppressed all PL-induced effects, including the activation of caspases, reducing apoptotic gene expression, and inhibition of cell survival signaling.

Activation of either the intrinsic apoptosis pathway or the extrinsic apoptosis pathway may cause the activation of effectors of various caspases, leading to cellular apoptosis. Caspases can be classified according to their function and activation as initiator caspases (caspases 2, 8, and 9) or executioner caspases (caspases 3, 6, and 7) [30]. Our results indicate that PL generates ROS and activates caspase-9 and caspase-3. However, caspase-8 is not involved. Caspase-9 is well known to be one of the key factors for the intrinsic pathway. These mechanisms were initiated by the generation of ROS, due to PL treatment. As shown in our study, pre-treatment with NAC could fully inhibit this cascade of events (Figure 5). Caspase-mediated pathways related to apoptosis were induced by PL in these cells, as confirmed by the addition of the Z-VAD-FMK pan-caspase inhibitor, which completely suppressed cell death induced by PL (Figure 3). Moreover, overexpression of Bcl-_XL_ and Bcl-2 can act against the cytotoxic effect of chemotherapeutic drugs, suggesting a potential role of these proteins in TC resistance to drug-induced cytotoxicity [31]. In this study, we also determined the decrease in Bcl-_XL_ expression in KMH-2 cells under PL treatment (Figure 3A), suggesting that PL treatment in human TC cells can induce cellular apoptosis through the intrinsic mitochondrial death pathway.

Previous research has indicated that PL is highly selective in targeting cancer cells, but not healthy cells [7]. Furthermore, a good safety profile has been observed in previous in vivo toxicological testing, showing high absorption through the gastrointestinal tract and >50% of bioavailability after oral administration of PL in mice [32]. The effects of PL on various molecular targets involved in cancer development and progression have been reported, focusing on its low toxicity and advanced pharmacokinetic features. However, owing to the lack of nanomolar potency and less soluble in water of PL, its applicability is presently limited. Pharmaceutical chemists may be able to derive compounds characterized by improved anticancer activity and appropriate drug-like physicochemical parameters. In addition, the use of modern drug delivery systems may improve the efficacy and solve issues related to water solubility [33]. Recently, great efforts have been made in to optimize PL activity through physicochemical parameters, pharmacokinetics, and safety, making PL a good candidate for future anticancer therapy.

Standard treatment for TC usually includes surgery, thyroid-stimulating hormone suppressive therapy, and radioactive irradiation (RAI) for the ablation of TC remnants [34]. However, approximately 5% of TC patients have metastasis, and there is currently a lack of effective drugs and limited efficacy of RAI for this condition. Several naturally occurring products, such as flavonoids, resveratrol, quercetin, catechins, myricetin, apigenin, and curcumin, have been investigated as having the potential to slow down or inhibit dedifferentiation and cancer progression [35]. Some human studies have also described the beneficial effects of plant-derived products with the potential for TC management and have found that different classes of flavonoids may have distinct effects in determining TC risk [36].

ROS-independent molecular targets of PL, such as PI3K/Akt/mTOR inhibition, have been reported [37]. PL also has anti-invasive properties, such as inhibiting the expression of Twist, N-cadherin, p120-ctn/vimentin/N-cadherin complex, IL-6, IL-8, MMP-9, and ICAM-1 [38]. PL also shows antiangiogenic effects by decreasing the expression of vascular endothelial growth factor protein [39]. Furthermore, PL works in concert with existing breast and prostate cancer treatments by increasing the efficacy of the chemotherapeutics 5-fluorouracil, cisplatin, doxorubicin, paclitaxel, and curcumin, increasing intracellular ROS levels, and enhancing the radiosensitivity of cancer cells [38,40,41]. ROS generation is strictly regulated in normal physiology; however, dysregulated ROS activation can cause cellular damage [42]. Because there is usually a higher level of ROS production in cancer cells than in normal cells, once intracellular ROS production is increased, cancer cells are more sensitive than normal cells, resulting in cellular damage [43,44]. Furthermore, exogenously generated ROS-induced oxidative stress is considered an effective therapy is owing to its selective effects against cancer cells, and PL has been reported to induce ROS in cancer cells, but not in normal cell lines [45,46]. Although enhancing intracellular ROS has been believed to be a new therapeutic strategy for multiple human cancers, related studies in TC remain scarce.

In this study, we found that PL significantly inhibited tumor growth in four TC cancer cell lines through G2/M phase arrest, in agreement with the findings of previous reports [12,19]. PL-induced ROS generation led to TC cell apoptosis by activating the Akt signaling pathway, thereby initiating caspase and PARP activation (Figure 7). Because these effects were fully abolished by the addition of the ROS scavenger NAC, as well as Akt inducer and dominant active Akt transfection, these results indicate that the antitumor activity of PL was primarily due to ROS generation (Figure 3, Figure 4, Figure 5, Figure 6 and Figure 7). Prolonged exposure to PL for 48 h enhanced the number of apoptotic cells, and the effect was completely reversed by co-exposure to NAC (Figure 3C). Inhibition of cell survival signaling by the Akt/mTOR pathway was also targeted by PL. In previous studies, PL has been shown to inhibit cell growth in various cancer cell lines, with an IC_50_ value of less than 10 μM, in agreement with the small micromolar doses observed here [12,13,14,15,19].

**Figure 9:**
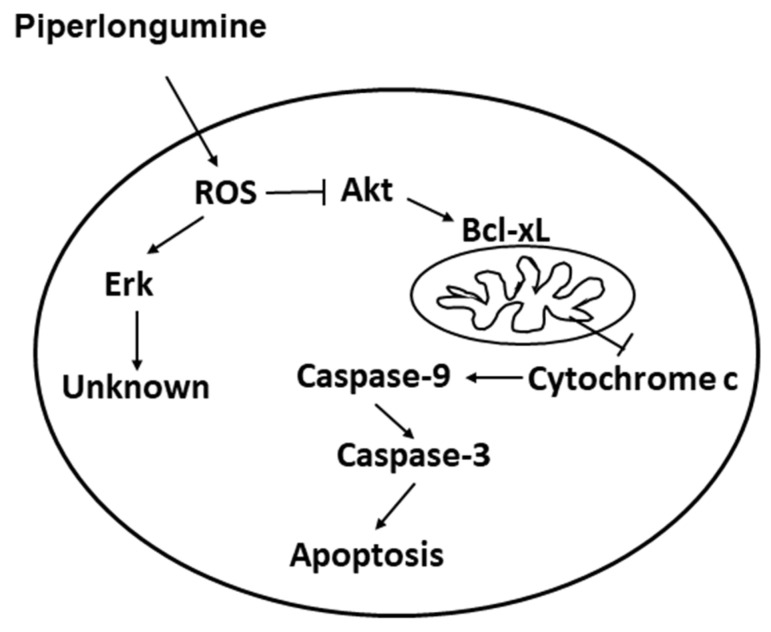
Illustration of the mechanisms involved in piperlongumine-mediated cellular apoptosis.

Human TC cell lines are frequently used for TC studies, with various lines originating from differentiated and undifferentiated human thyroid tumors. They have maintained hallmarks of cancer cells and are pure, genetically identical, and easily cultured, and can be genetically operated [47]. The cell line originates from clinical specimens and is generated by the selection of the resistance or rapidly proliferating cells during passages. The IHH-4 line used in this study was derived from a patient with cervical lymph node metastasis; it possesses multidrug resistance in PTC, accounting for approximately 80% of all TCs reported. The KMH-2 and 8505c lines are derived from ATC, which is undifferentiated, grows rapidly in elderly patients, and is not sensitive to most therapies [48]; as such, it is often fatal. PL showed great inhibitory effects against all three cell lines, suggesting broad applicability in TC treatment. In addition, WRO cells seemed more resistant to PL treatment than IHH-4, KMH-2, and 8505c cells (Figure 1). Because the PL-mediated anticancer effect in this study is through ROS and downstream Akt signaling (Figure 9), we also detected ROS induction in WRO cells under PL treatment. We demonstrated that higher doses of PL are required for raising ROS levels in WRO cells than in IHH-4, KMH-2, and 8505c cells (Figure 4A and Figure S1).

Cell proliferation and cell survival are modulated with an intricate network, such as signaling pathways; controlling one or two factors of them may not be enough to manage malignant growth. Because of the increased metabolism in malignant cells, ROS accumulation is common, and can be used as a therapeutic target for malignant cells. In the present study, we demonstrated that PL successfully induces ROS overexpression in TC cells to interfere with cell survival mechanisms and achieve apoptosis induction.

## 4. Materials and Methods

### 4.1. Cell lines and Cell Culture

The human multidrug-resistant PTC cell line (IHH-4) and ATC cell lines (KMH-2 and 8505c) were purchased from the Japan Collection of Research Bioresources Cell Bank (JCRB). The FTC cell line (WRO) was provided by Prof. Jen-Der Lin [49]. IHH-4 and KMH-2 cells were cultured with Dulbecco’s minimal essential medium (DMEM) + Roswell Park Memorial Institute (RPMI) (1:1) medium (Gibco, Gaithersburg, MD, USA); 8505c cells were maintained with MEM (Gibco); and WRO cells were incubated in RPMI 1640 medium (GIBCO) supplemented with 10% fetal bovine serum (Biological Industries, Kibbutz Beit Haemek, Israel), at 37 °C in a 5% CO_2_ incubator. The passage ranges for the cell lines used in this study were p05–p20 post-purchase.

### 4.2. Cell Viability Assay

The cells (5 × 10^3^ cells/well) were seeded in a 96-well cell culture dish, and the plate containing the previous medium was incubated. After overnight incubation, the cells were administrated with the control medium (containing 0.01% DMSO) or PL (Cayman, MI, USA). After administration for 24 and 48 h, cell survival was detected with a CCK-8 assay kit (Enzo Life Sciences, Farmingdale, NY, USA). Three independent assays were performed.

### 4.3. Colony Formation Assay

The cells were cultured in 6-well plates (10^3^ cells/well), and the plates were cultured at 37 °C with the previous medium. And the cells were administrated with DMSO or PL. After 12 days of incubation, the colony of the cells and the colony morphology were detected under 10% crystal violet (Sigma-Aldrich, St. Louis, MO, USA) staining. The colony size and the colony number were examined.

### 4.4. Cell Cycle Analysis

Cells (1 × 10^6^ cells/dish) were cultured in a 10 cm culture plate. After overnight cell attachment, the cells were administrated with DMSO or PL with various durations. The process for cell cycle analysis was referred to as previously reported [4,50,51]. Three independent assays were performed.

### 4.5. Cell Death Analysis

Cells were treated with DMSO or PL for various durations. The process for determining apoptosis with flow cytometry was referred to as previously reported [52]. The protein expressions involved in the mechanisms that PL-mediated apoptosis were examined with Western blots. caspase-3, caspase-8, caspase-9, anti-PARP, and anti-GAPDH antibodies were purchased from Cell Signaling Inc. (Danvers, MA, USA). GAPDH was used as a loading control. Z-VAD-FMK, a pan-caspase inhibitor, was purchased from ApexBio Technology LLC (Houston, TX, USA). The inhibitor of ROS, NAC, was purchased from Cayman. Moreover, the MAPK and Akt signaling pathways, including the total forms and phosphorylated forms of ERK, JNK, p38, Akt, and mTOR (all antibodies were purchased from Cell Signaling), were examined in the cells after PL treatment. PD98058 and perifosine, inhibitors of Erk and Akt, respectively, were purchased from LC Laboratories (Woburn, MA, USA). The plasmid, including pBSSK^+^ and constitutively active human Akt1 construct (pmAkT), were used as previously reported [50]. Three independent assays were performed.

### 4.6. Measurement of Cellular ROS

Cells (5 × 10^5^/dish) were treated with DMSO or PL for various durations. The cells were stained with dichlorofluorescin diacetate (DCFH-DA, 10 μM; Sigma-Aldrich) at 37 °C for 30 min; they were then washed three times with ice-cold phosphate-buffered saline (PBS). The fluorescence expressed by cells was measured by flow cytometry (Becton Dickinson, San Diego, CA, USA). In the ROS blocking experiments, cells were pre-treated with NAC for 2 h.

### 4.7. Western Blotting

Cells were treated with DMSO or PL for various durations and were lysed with the M-PER^TM^ protein extraction reagent (Thermo Fisher Scientific Inc., Rockford, IL, USA) with a 0.1% protease inhibitor cocktail. The sample containing total protein was loaded, using sodium dodecyl sulfate-polyacrylamide gel electrophoresis gels to separate proteins and then transferred the proteins on polyvinylidene fluoride membranes. The proteins were detected after blocking with a primary antibody and a secondary antibody. The experimental process could refer to our previous paper [53]. All original western blots are included in Figures S2–S75.

### 4.8. Xenograft Study

Six-week-old female nude mice (BALB/cAnN.Cg-Foxn1nu/CrlNarl) were purchased from the National Laboratory Animal Center. Mice were maintained in the animal facility of National Chiayi University, Chiayi, Taiwan. Because PTC is common in TC patients, we used a multidrug-resistant PTC cell line (IHH-4) to examine the anticancer activity of PL in vivo. IHH-4 cells (2 × 10^6^ cells/mice) were subcutaneously injected into the nude mice in the right flank. When the tumor growth was less than 1 mm^3^, the mice were divided into three groups of five animals each, and were then administrated with DMSO or PL. In the experimental group, mice received injections with PL (containing 5 and 10 mg/kg) on days 1, 3, 5, 7, 9, and 11. The mice received an injection of DMSO to be the control group. The tumor size was also measured on these days. The tumor volume was estimated using the following equation: *L* × *S*^2^/2, where *L* is the longest diameter, and *S* is the shortest diameter). After treatment for 11 days, mice were sacrificed, and tumor weights were determined. The experimental protocol complied with Taiwan’s Animal Protection Act and was approved by the Laboratory Animal Care and Use Committee of the National Chiayi University (IACUC Approval No. 102021).

### 4.9. Immunohistochemical Staining and TUNEL Assay of Tissue Sections

The tumor tissues were fixed with formalin (Sigma-Aldrich) at 4 °C for 24 h, and soaked with 100% alcohol for 5 min. Specimens were embedded with paraffin after incubation with xylene. Finally, the specimens were sectioned into 4 µm slices. The specimens were stained with HE (Sigma-Aldrich) for 15 min at room temperature, and the stained specimens were visualized under a microscope. In addition, the specimen slices were stained by the terminal deoxynucleotidyl transferase-mediated nick end labeling (TUNEL) assay with the DeadEnd™ Colorimetric Apoptosis Detection System (Promega, Madison, WI, USA) referring to the manufacturer’s instructions. Briefly, the slides were incubated by equilibration buffer for 10 min, and treated with proteinase K for 10 min. The samples were washed with PBS and treated with TdT enzyme at 37 °C for 1 h. The slides were then incubated with horseradish peroxidase-labeled streptavidin, and detected with diaminobenzidine. Finally, the images of the specimens were examined under a microscope.

### 4.10. Statistical Analysis

Data are shown as the mean ± SD in the experiments. Differences between the control group and test groups were analyzed with a one-way analysis of variance and Fisher’s least significant difference test. In the in vivo mice study, the Mann–Whitney *U*-test was used. Statistical significance was defined at *p* < 0.05, < 0.01, or < 0.001 in the statistical tests.

## 5. Conclusions

Our findings demonstrate that PL exhibits antitumor activity against TC cell lines, including ATC and multiple drug-resistant PTC and FTC cells, by inhibiting cell proliferation, enhancing G2/M phase arrest, and promoting apoptosis. In addition, PL can trigger TC cell death through intrinsic cellular apoptosis by ROS induction and inhibition of downstream Akt signaling (Figure 9). This is the first study to report that TC cell lines are sensitive to PL. The study also investigated the anti-TC mechanisms of PL in detail, which suggested that it is a potential chemotherapeutic agent for TC treatment.

## Figures and Tables

**Table 1:** IC_50_ values of piperlongumine in human thyroid cancer cells.

	Time (h)	Cell Line			
		IHH-4	WRO	8505c	KMH-2
Piperlongumine (μM)	24	3.2	12.52	3.3	2.4
	48	2.8	5.58	2.8	1.7

## Data Availability

Data is contained within the article or supplementary material.

## Supplementary Materials

The following are available online at https://www.mdpi.com/article/10.3390/cancers13174266/s1, Figure S1: Modulation of cellular ROS with piperlongumine (PL) treatment in WRO cells., Figures S2–S75: Original western blots.
