# Supplementary material for: Piperlongumine, a Potent Anticancer Phytotherapeutic, Induces Cell Cycle Arrest and Apoptosis In Vitro and In Vivo through the ROS/Akt Pathway in Human Thyroid Cancer Cells
Source: Cancers (Basel). 2021 Aug 24;13(17):4266. doi: 10.3390/cancers13174266 (PMC8428232; doi:10.3390/cancers13174266)
Supplement: Supplementary file 1 [file cancers-13-04266-s001.zip › cancers-1320686-supplementary.pdf]

Supplementary Materials: Piperlongumine, a Potent Anticancer Phytotherapeutic, Induces Cell Cycle Arrest and Apoptosis In Vitro and In Vivo through the ROS/Akt Pathway in Human Thyroid Cancer Cells

Fang-Ping Kung, Yun-Ping Lim , Wen-Ying Chao , Yi-Sheng Zhang , Hui-I Yu , Tsai-Sung Tai, Chieh-Hsiang Lu, Shu-Hsin Chen, Yi-Zhen Li, Pei-Wen Zhao, Yu-Pei Yen and Ying-Ray Lee

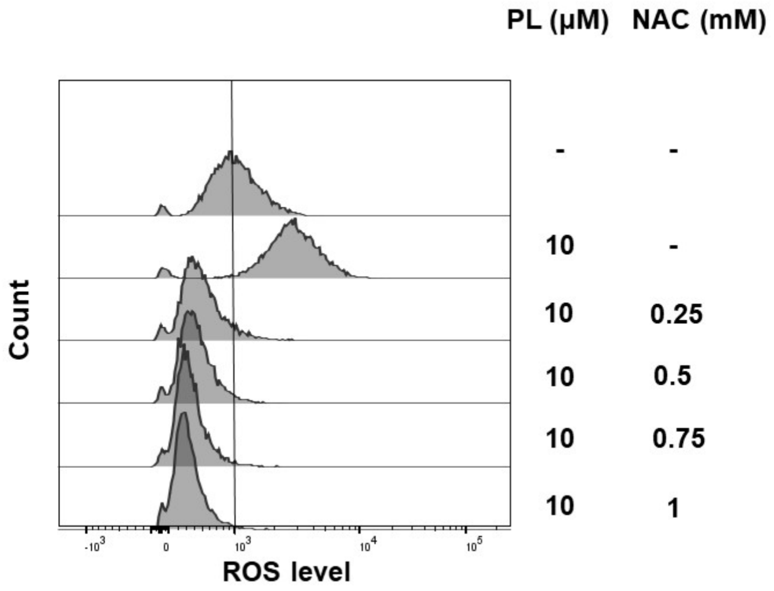

Figure S1. Modulation of cellular ROS with piperlongumine (PL) treatment in WRO cells. WRO cells were incubated with PL, and NAC was used to inhibit ROS activation. The expression of ROS was determined with fluorescence-activated cell sorting (FACS) flow cytometry.

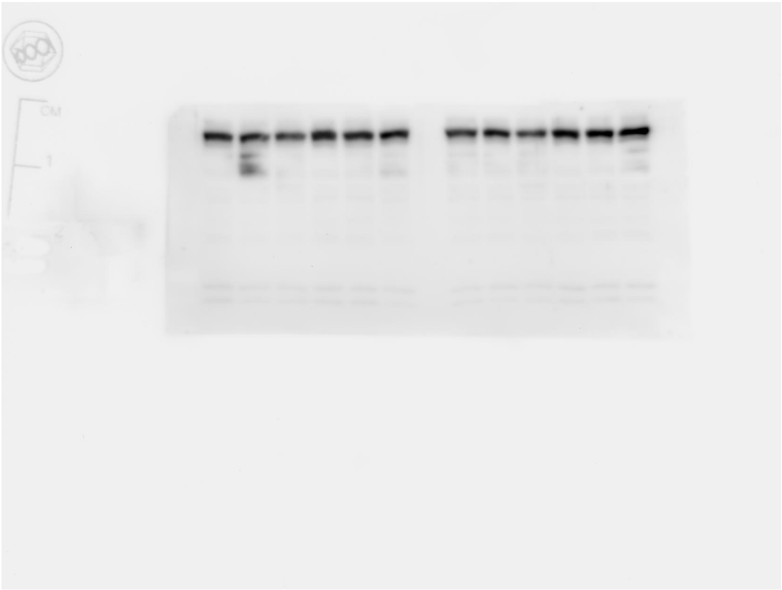

Figure S2. IHH-4/caspase-8.

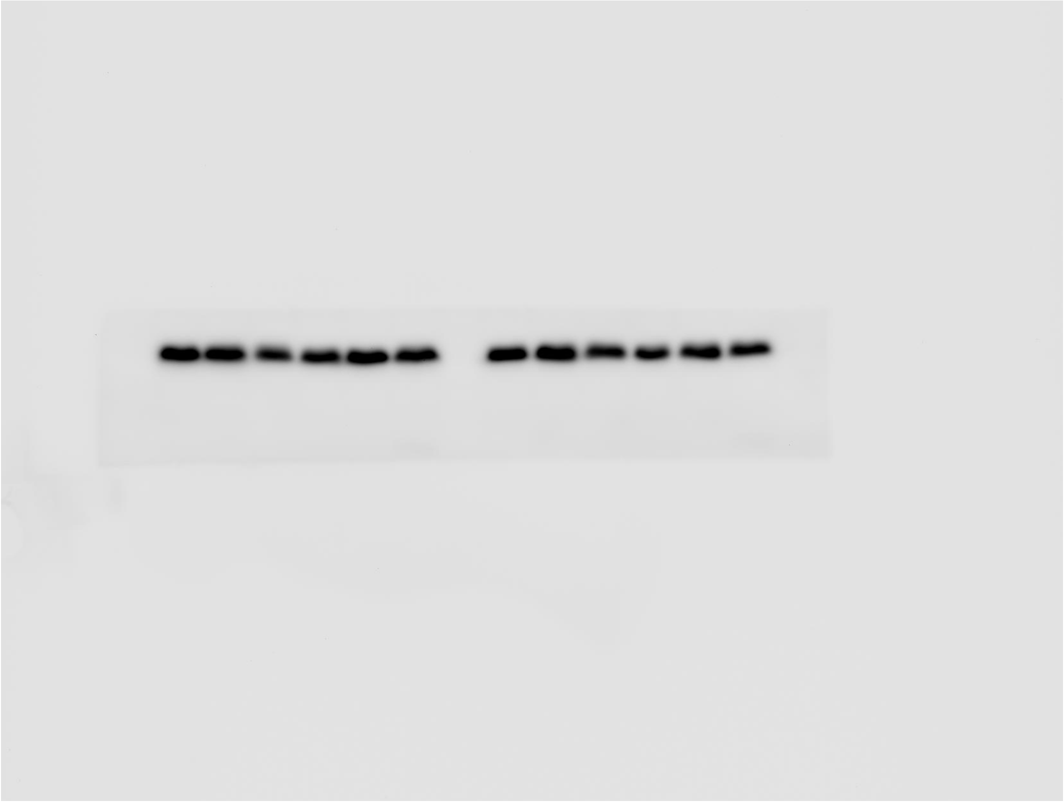

Figure S3. IHH-4/Bcl-xl.

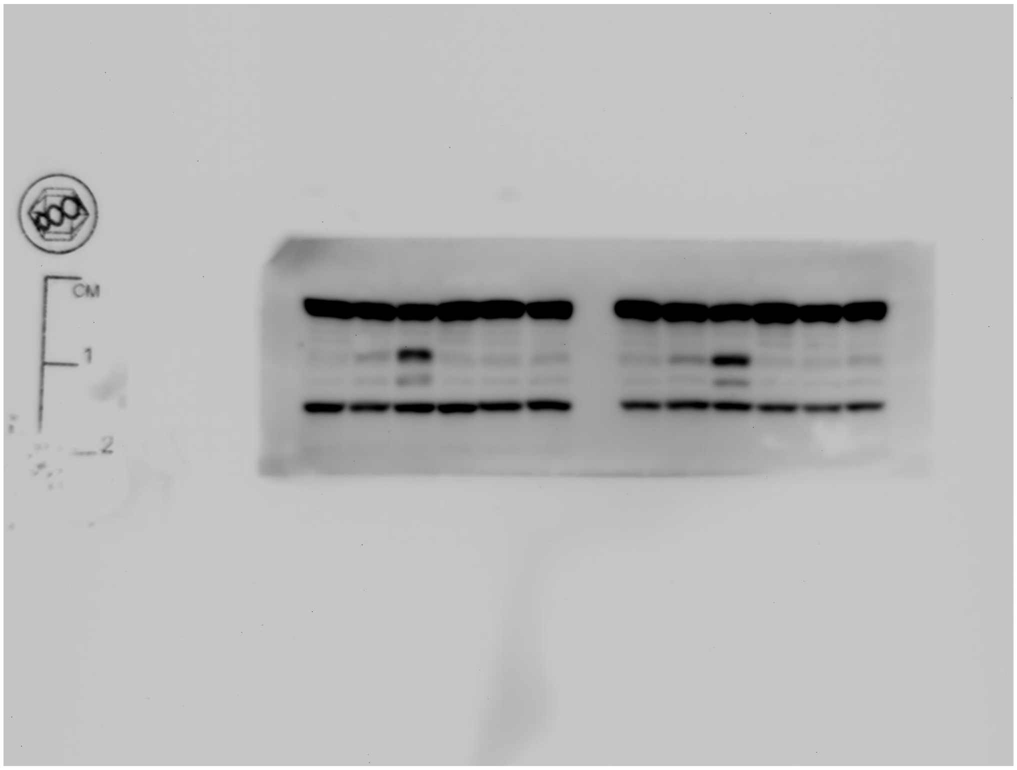

Figure S4. IHH-4/caspase-9.

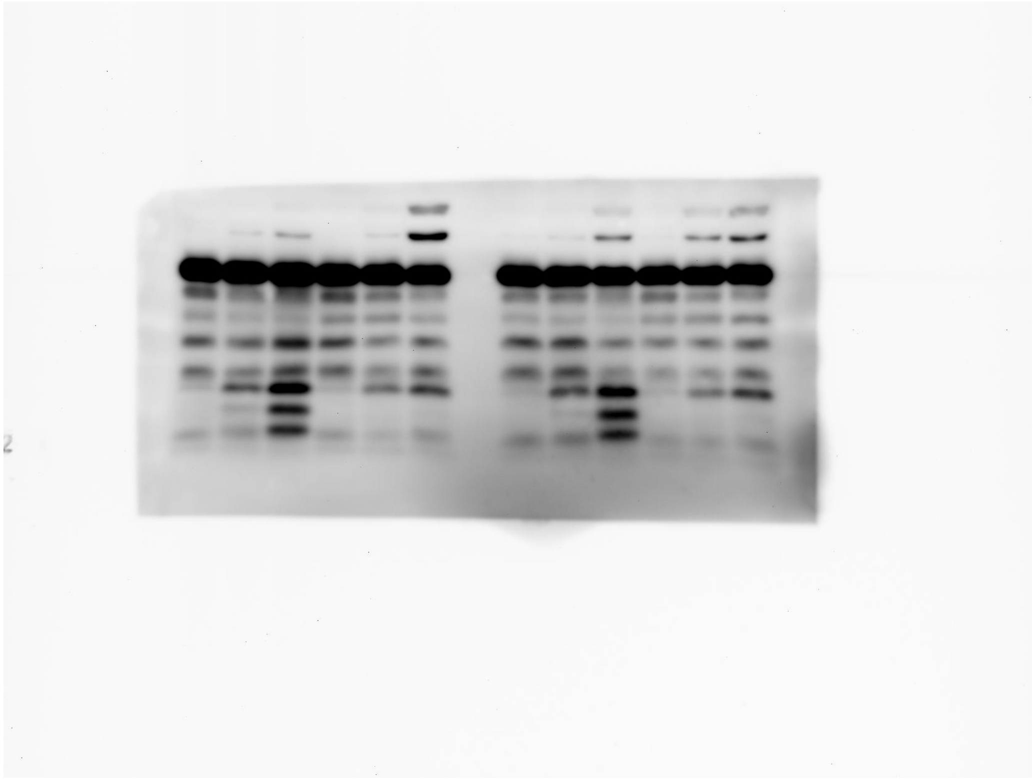

Figure S5. IHH-4/caspase-3.

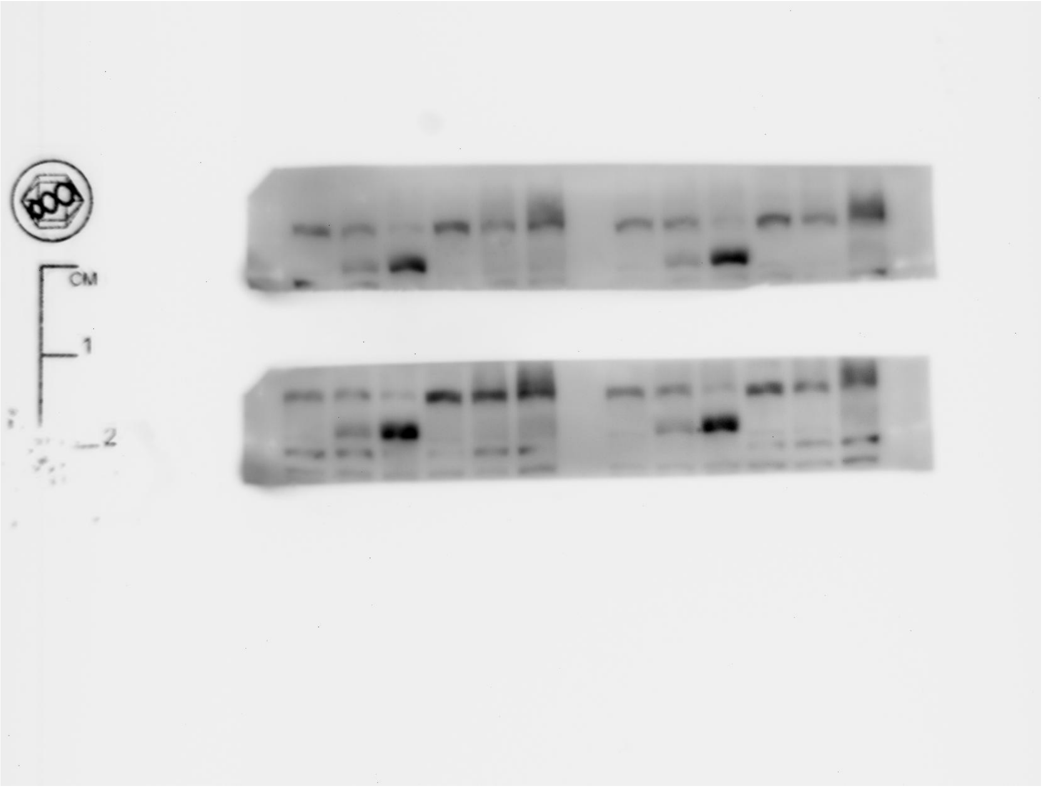

Figure S6. IHH-4/PARP-1.

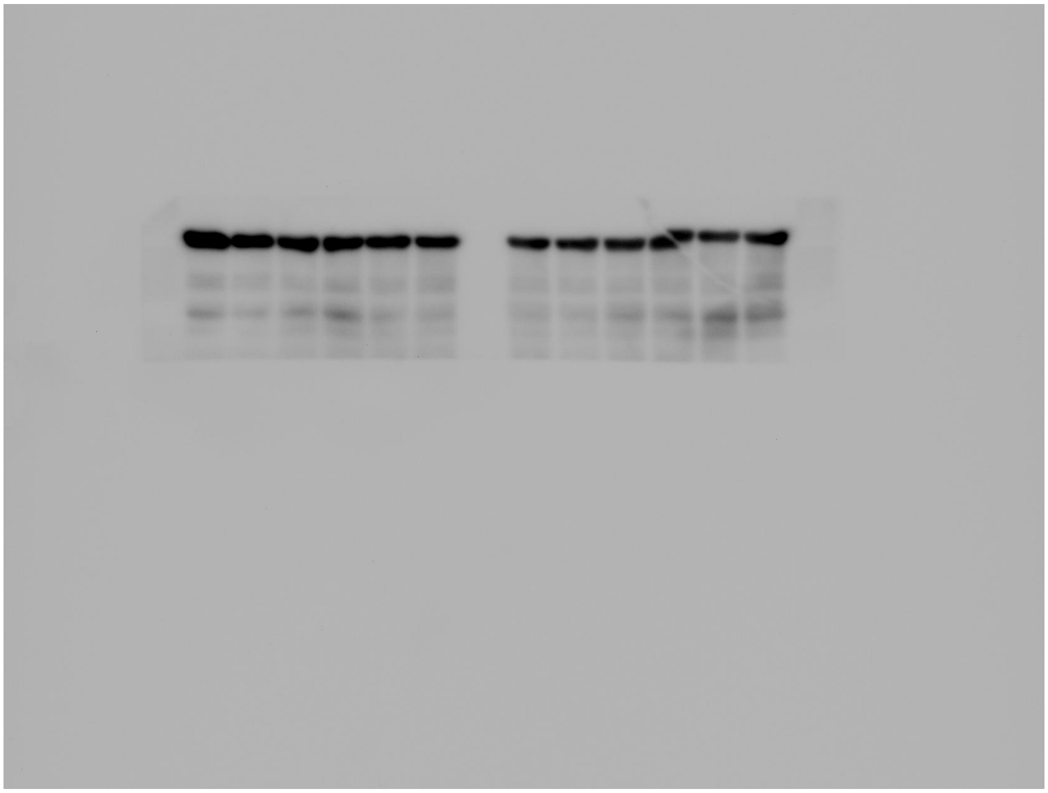

Figure S7. IHH-4/GAPDH.

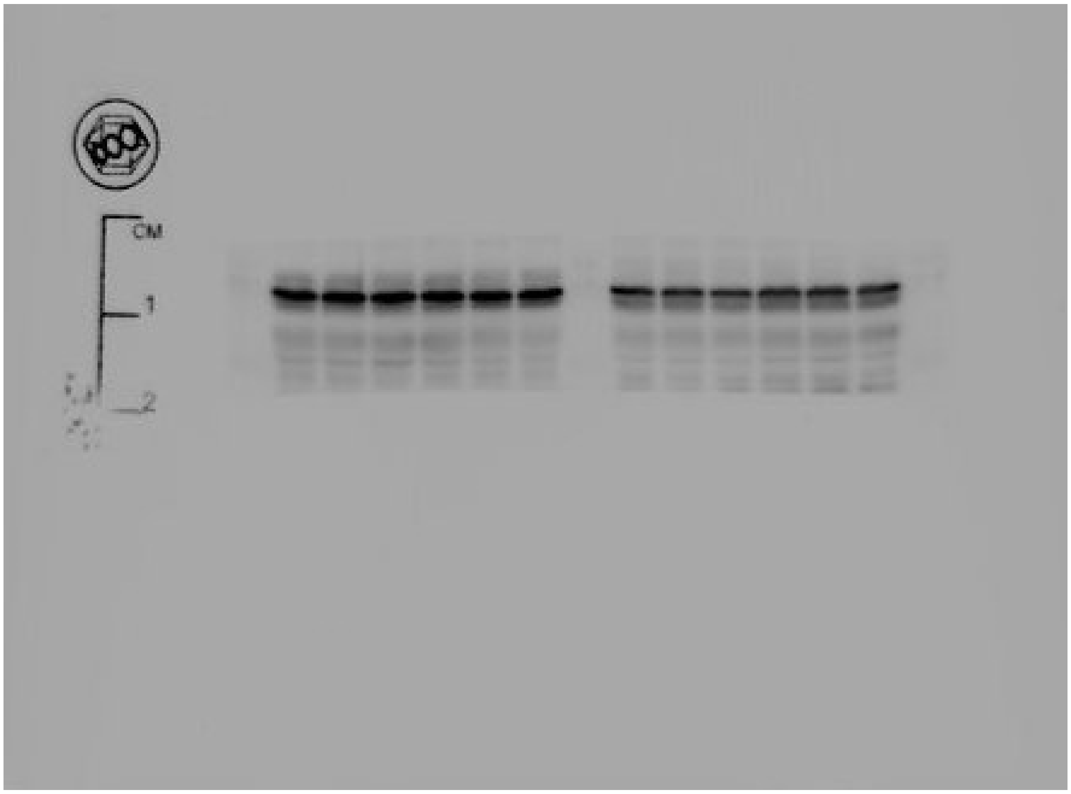

Figure S8. 8505c/caspase-8.

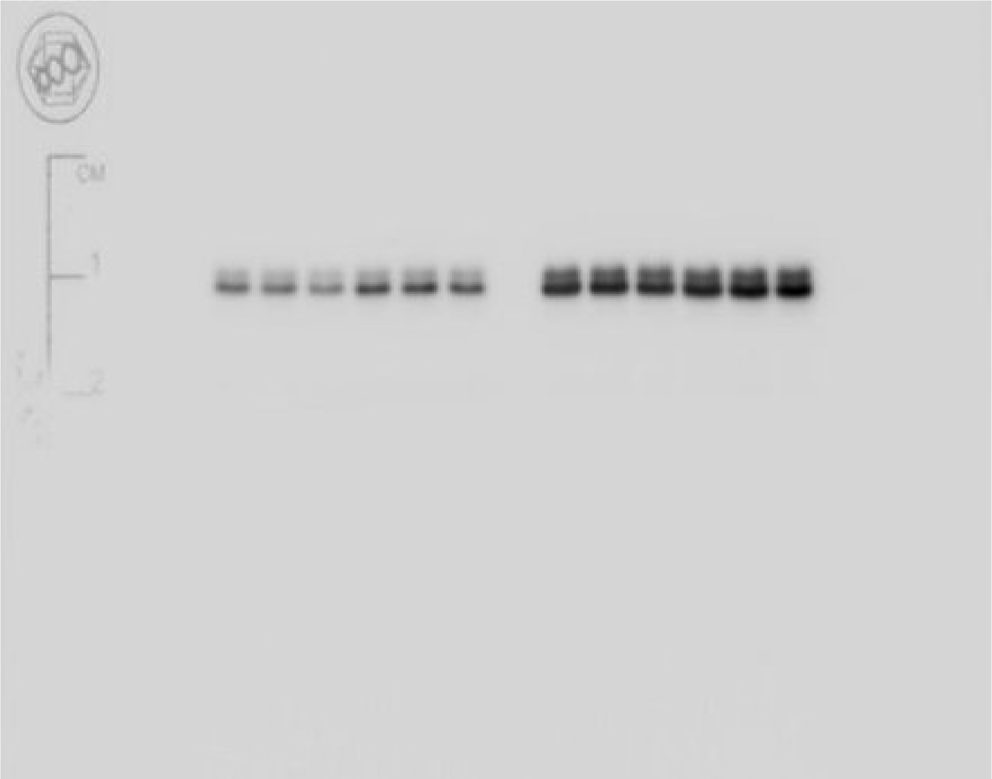

Figure S9. 8505c/Bcl-xl.

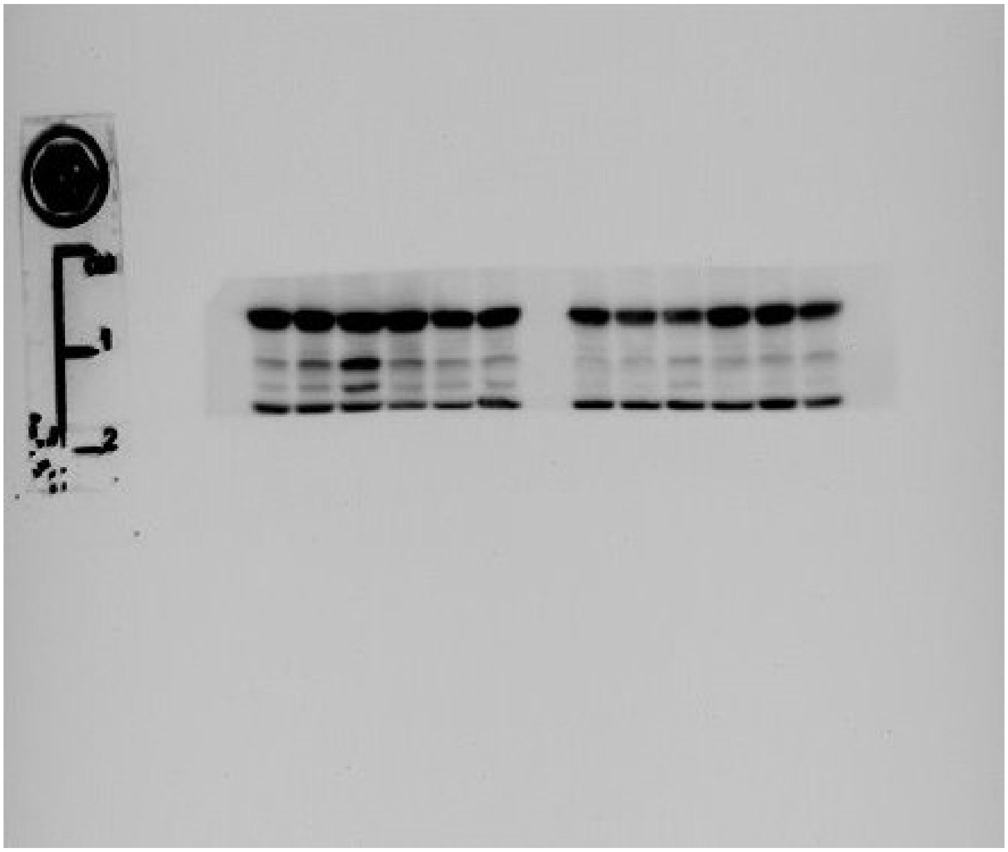

Figure S10. 8505c/caspase-9.

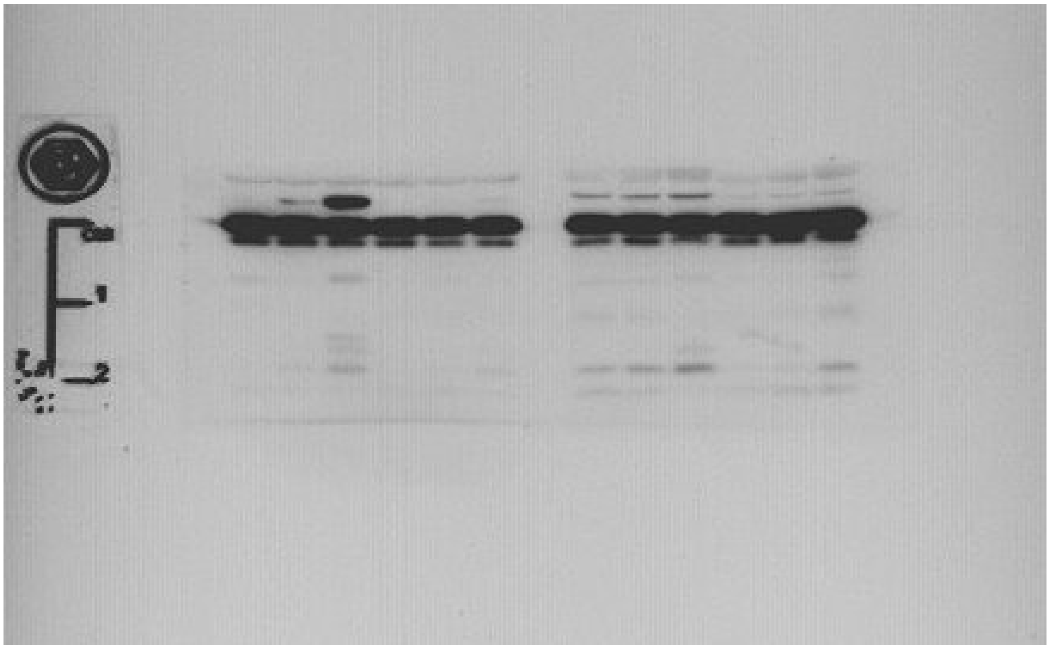

Figure S11. 8505c/caspase-3.

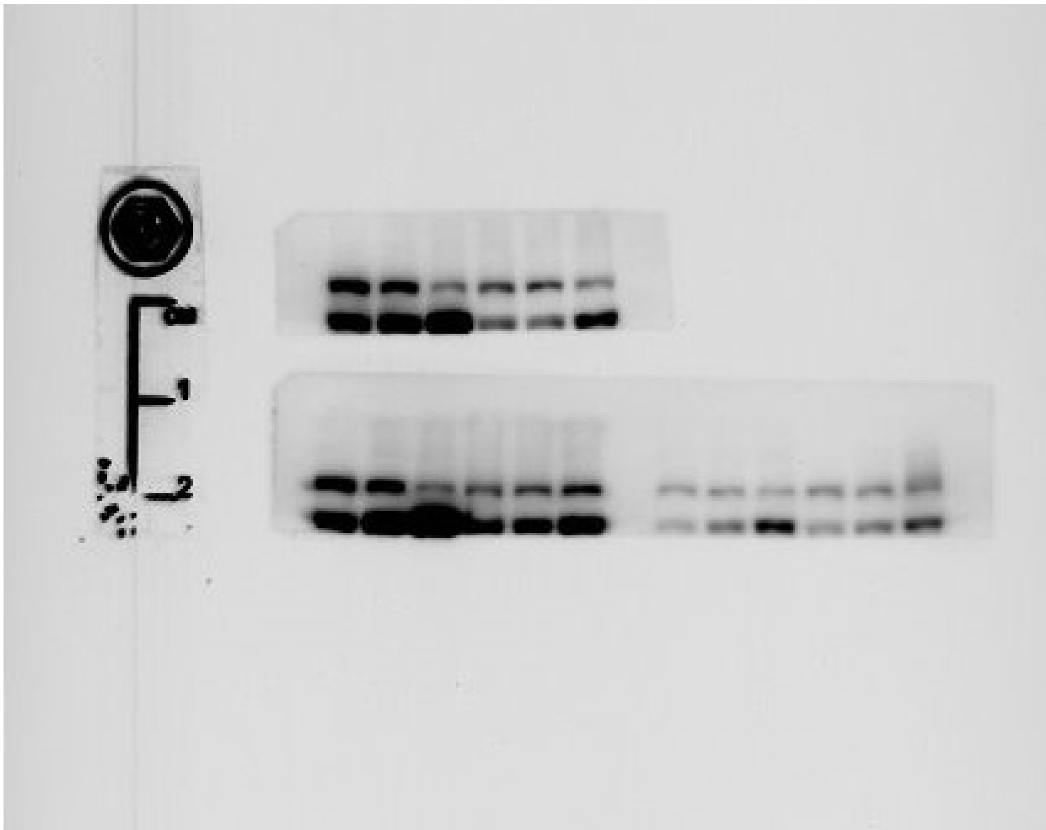

Figure S12. 8505c/PARP-1.

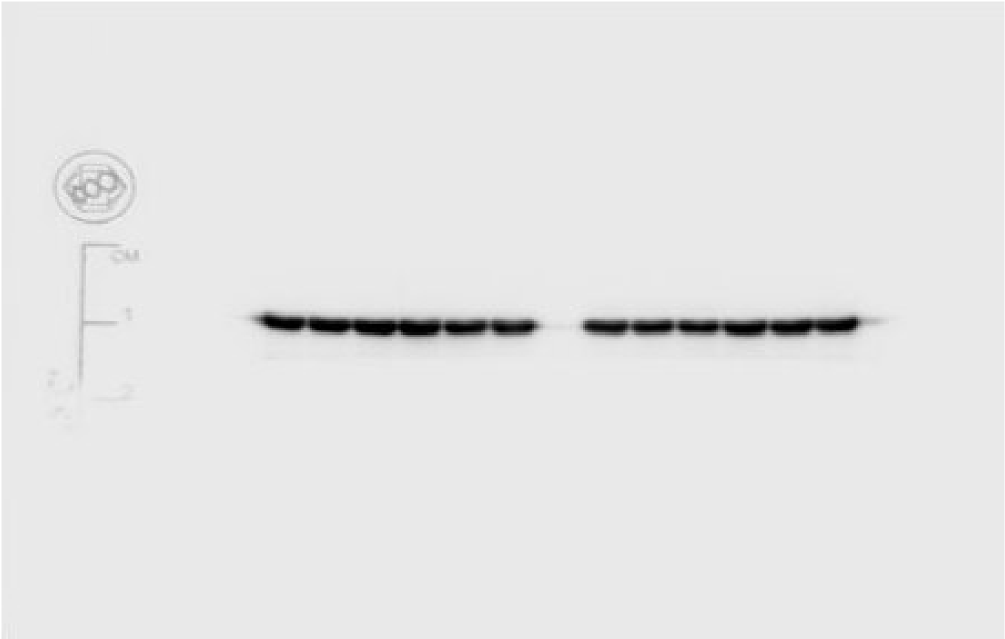

Figure S13. 8505c/GAPDH.

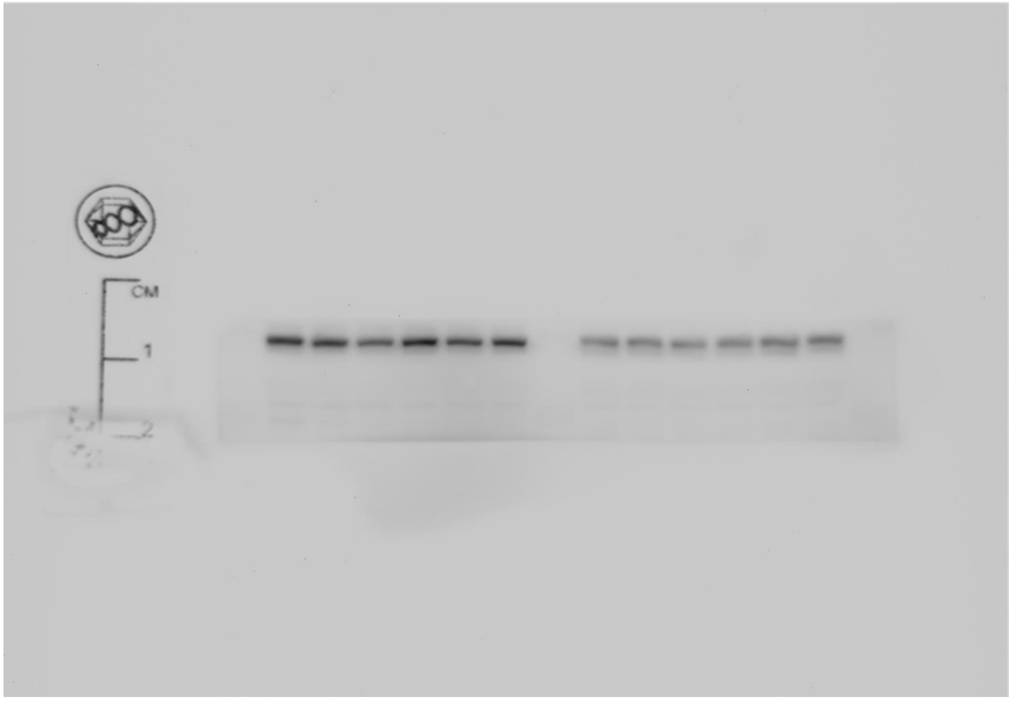

Figure S14. KMH-2/caspase-8.

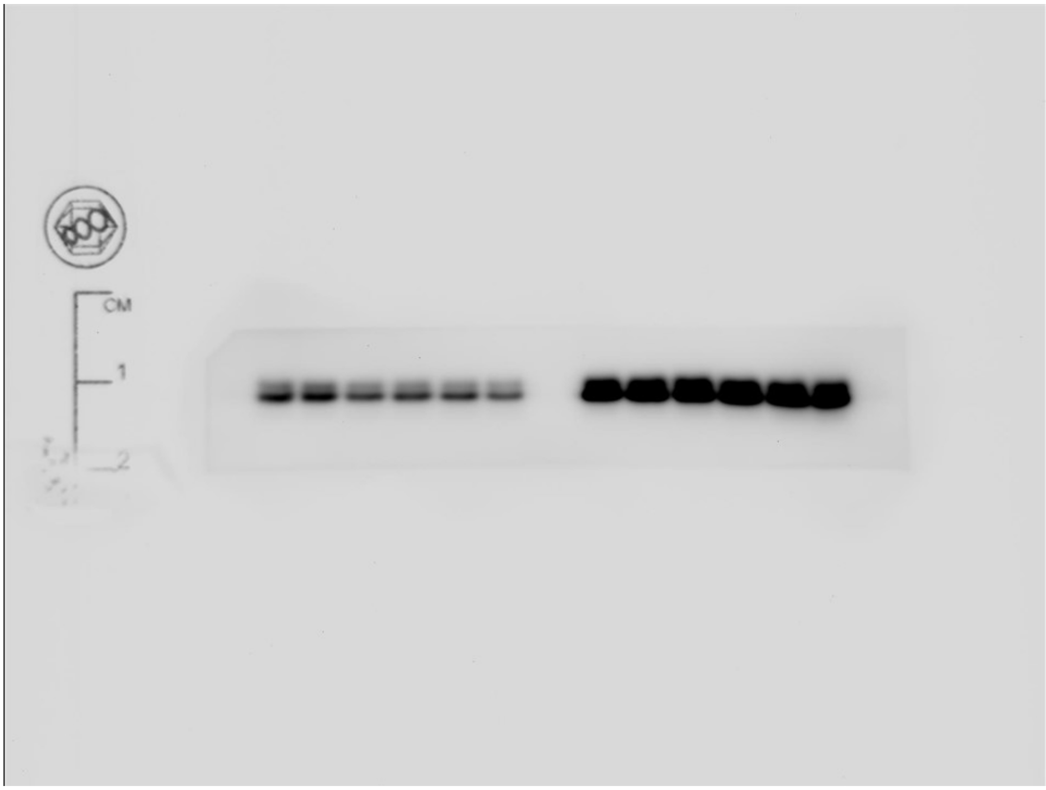

Figure S15. KMH-2/Bcl-xl.

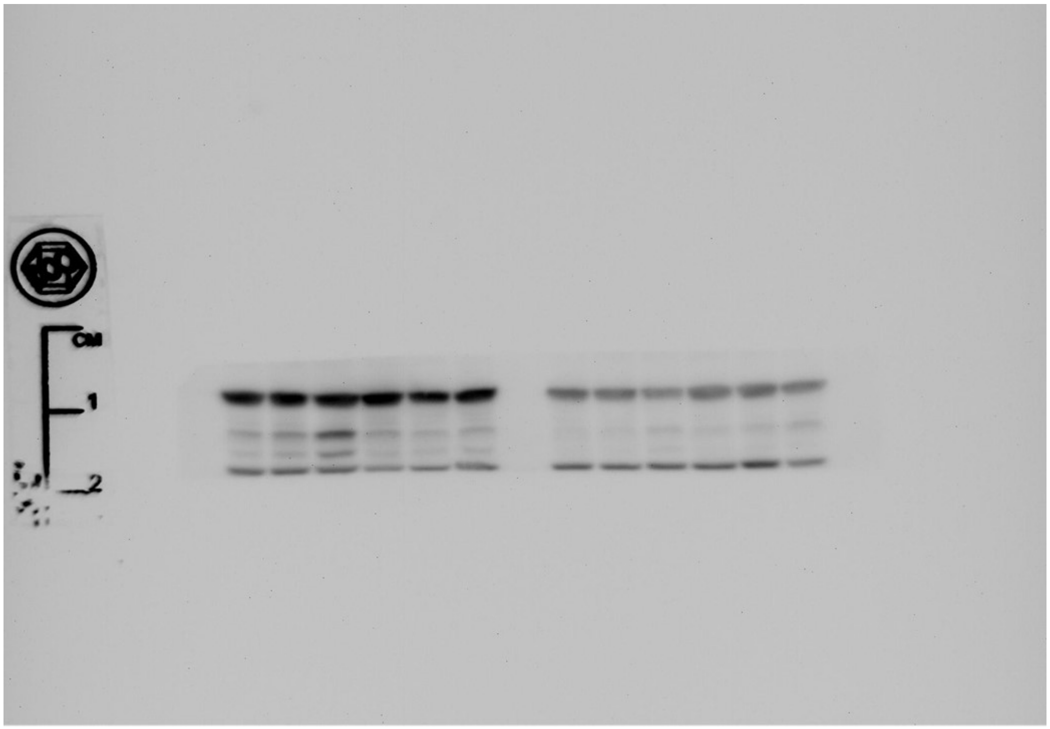

Figure S16. KMH-2/caspase-9.

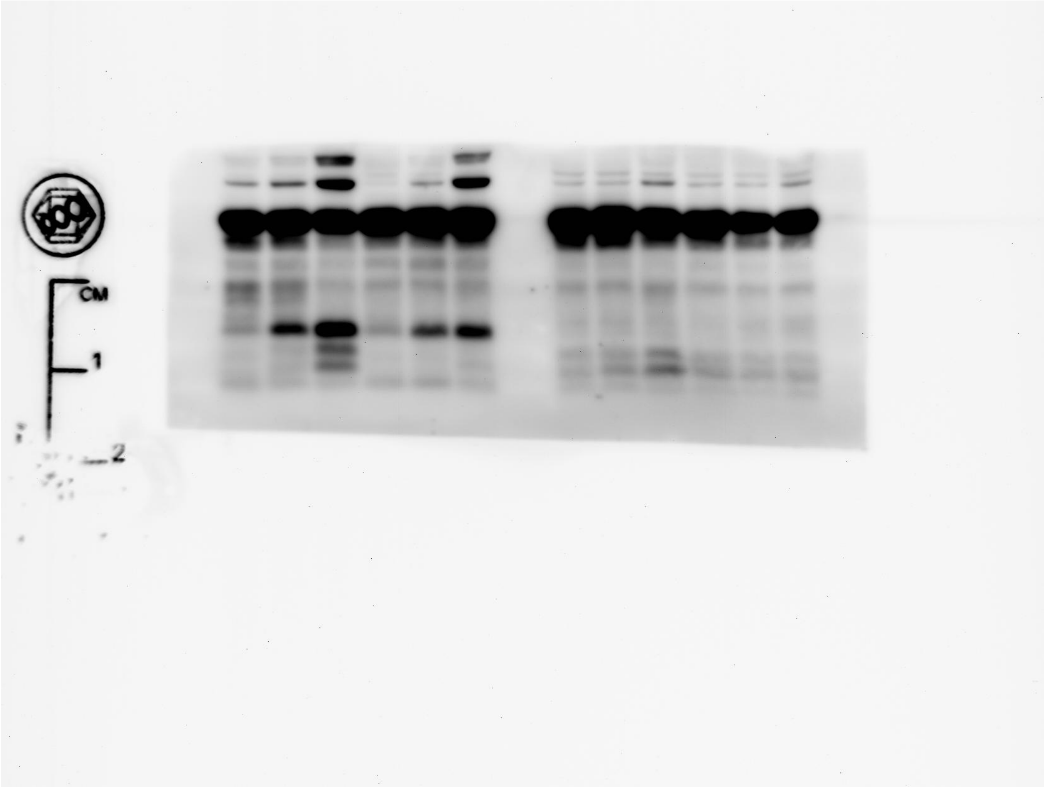

Figure S17. KMH-2/caspase-3.

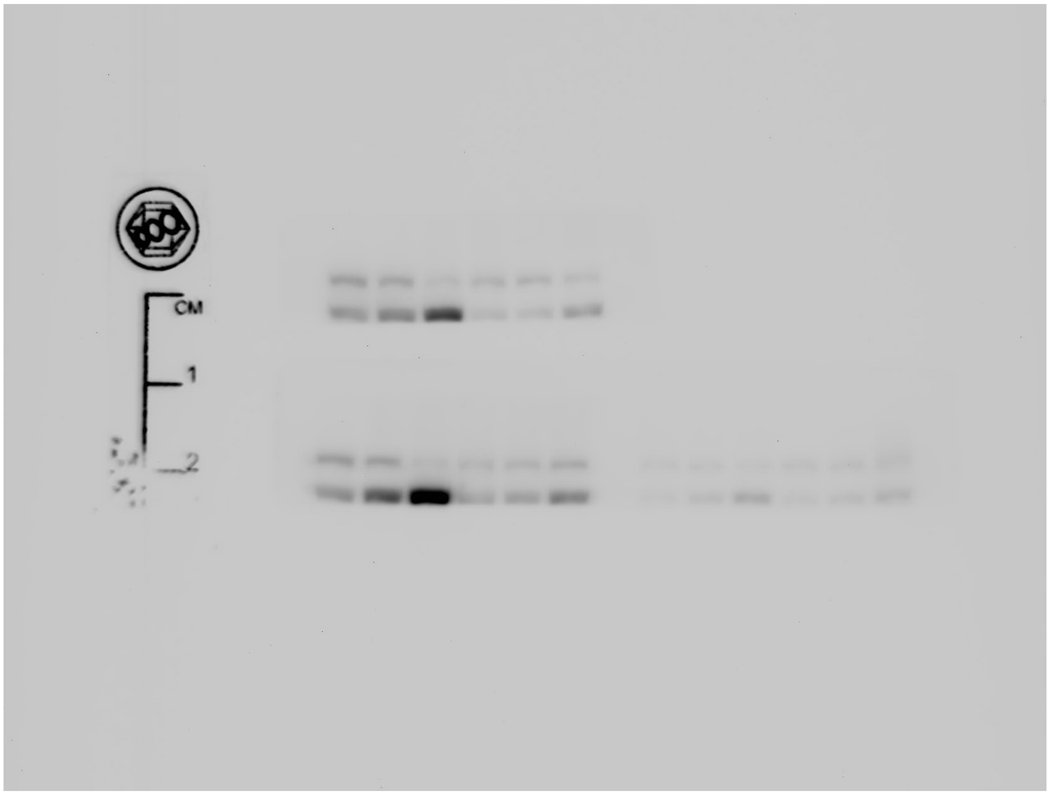

Figure S18. KMH-2/PARP-1.

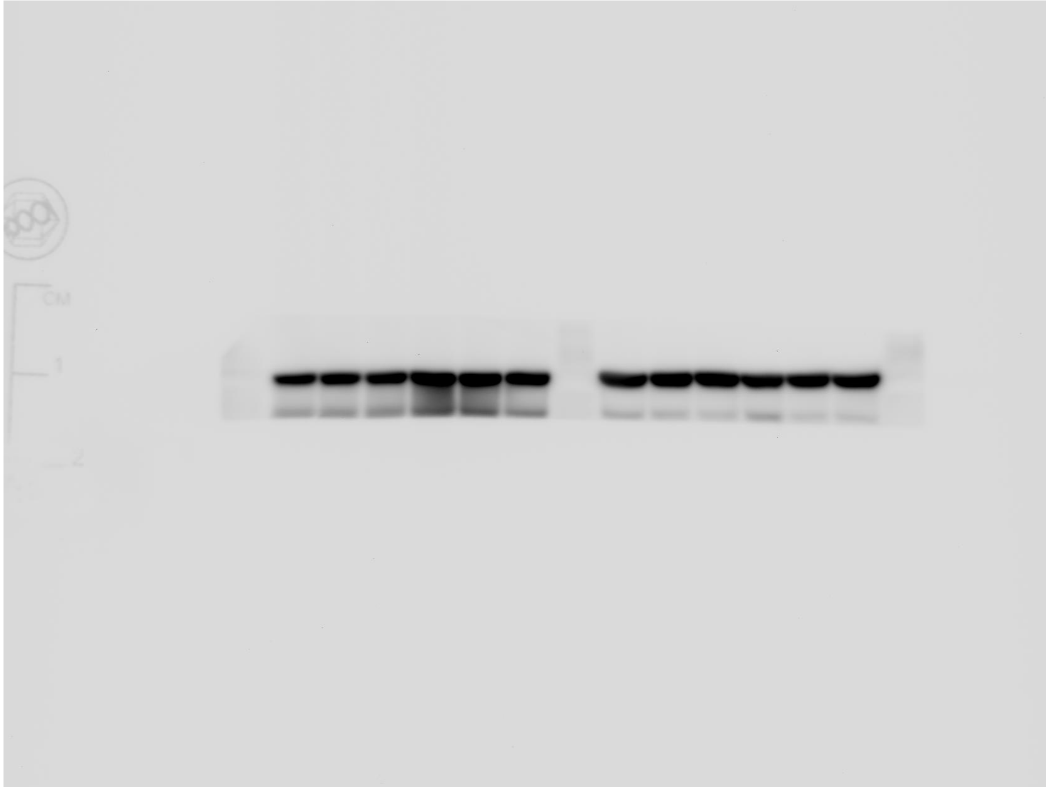

Figure S19. KMH-2/GAPDH.

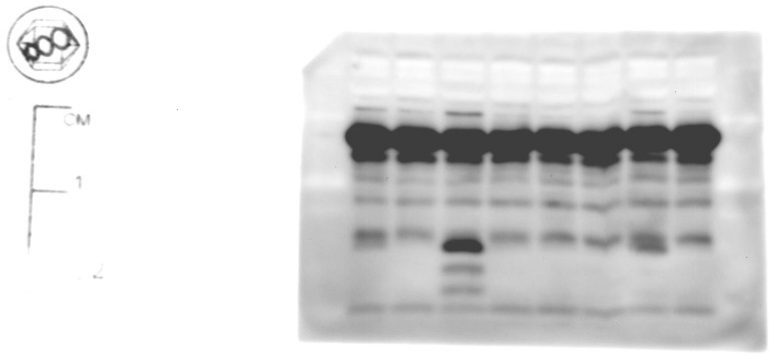

Figure S20. IHH-4/caspase-3.

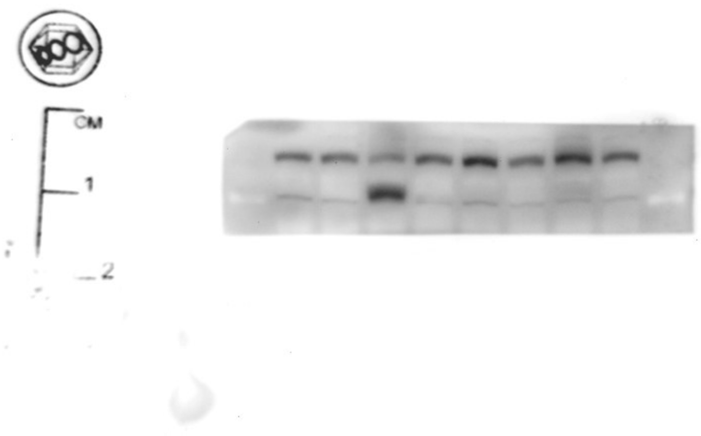

Figure S21. IHH-4/PARP-1.

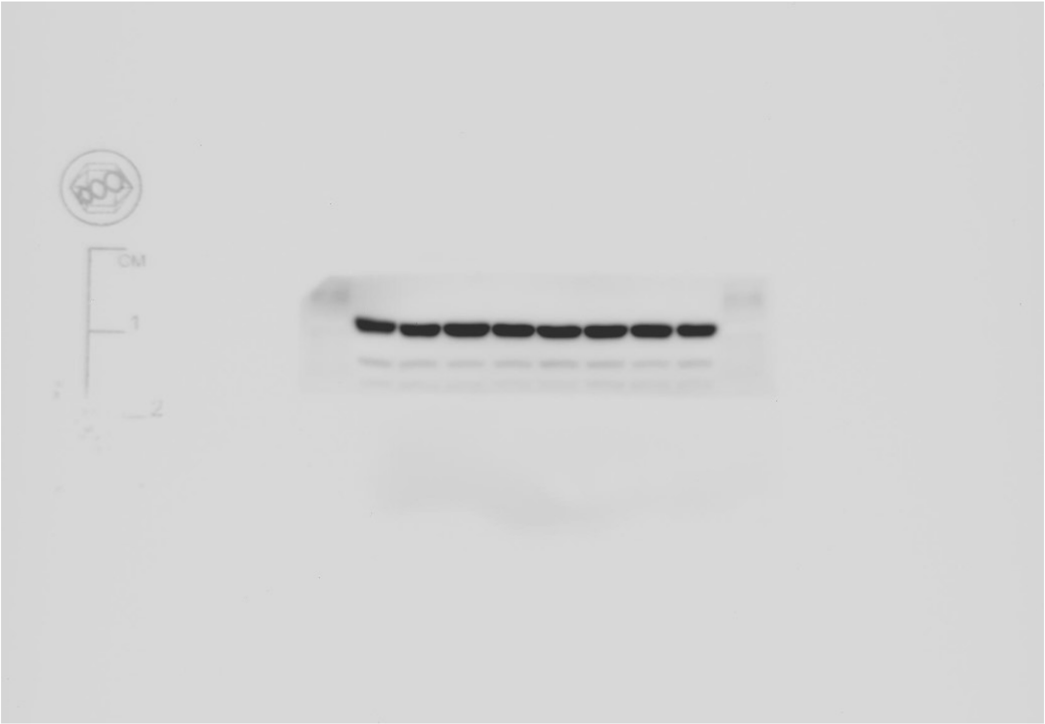

Figure S22. IHH-4/GAPDH.

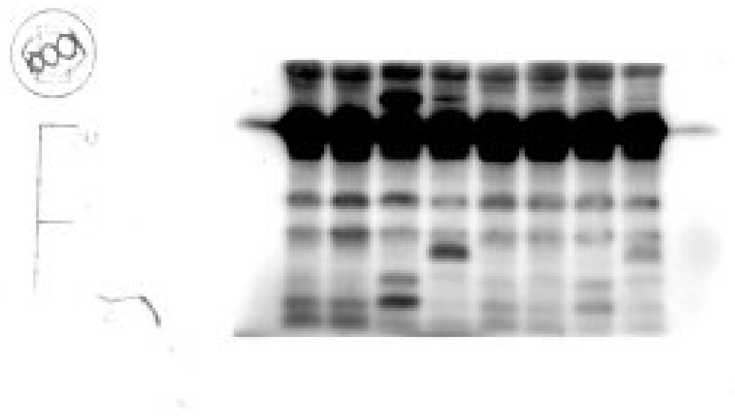

Figure S23. 8505c/caspase-3.

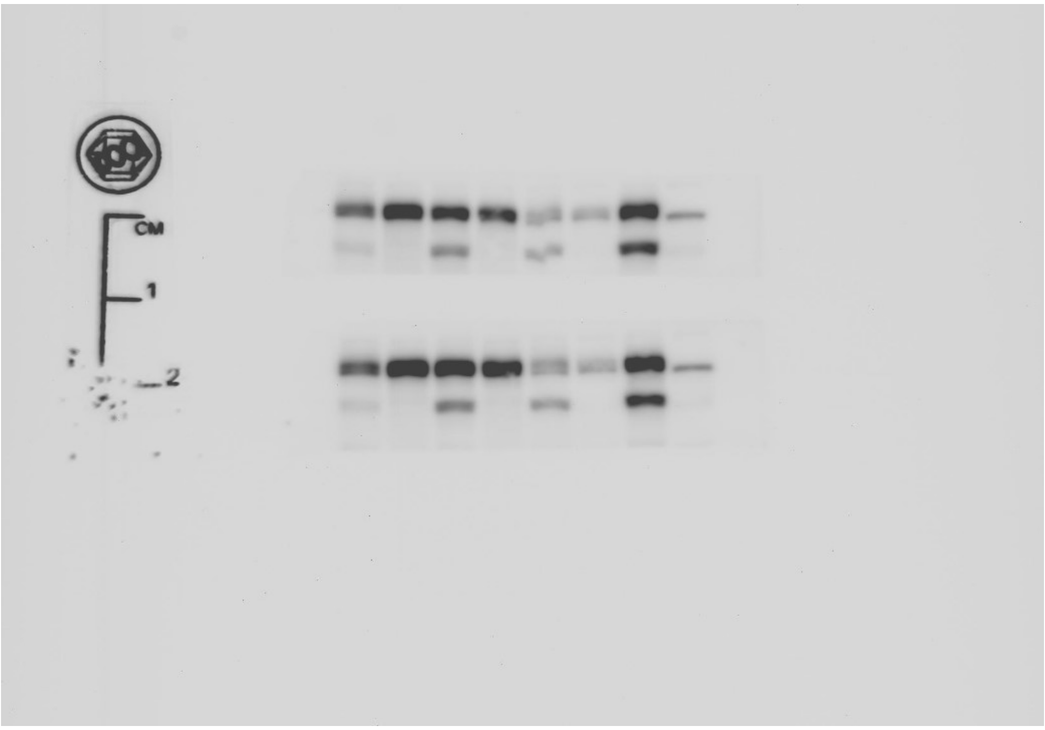

Figure S24. 8505c/PARP-1.

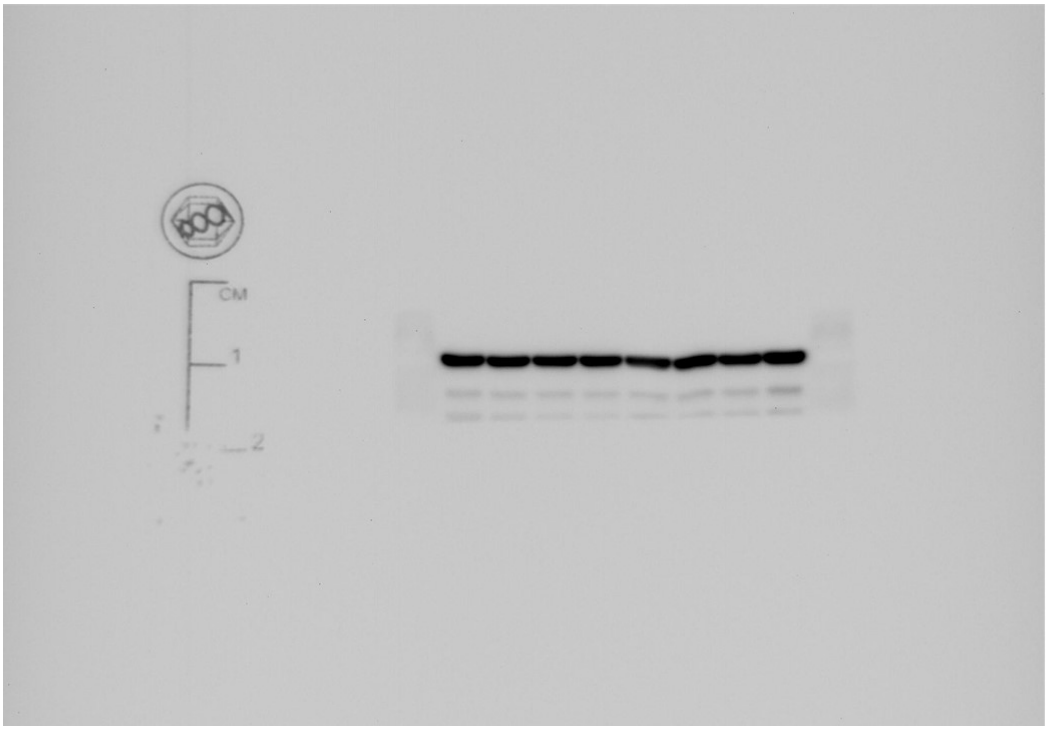

Figure S25. 8505c/GAPDH.

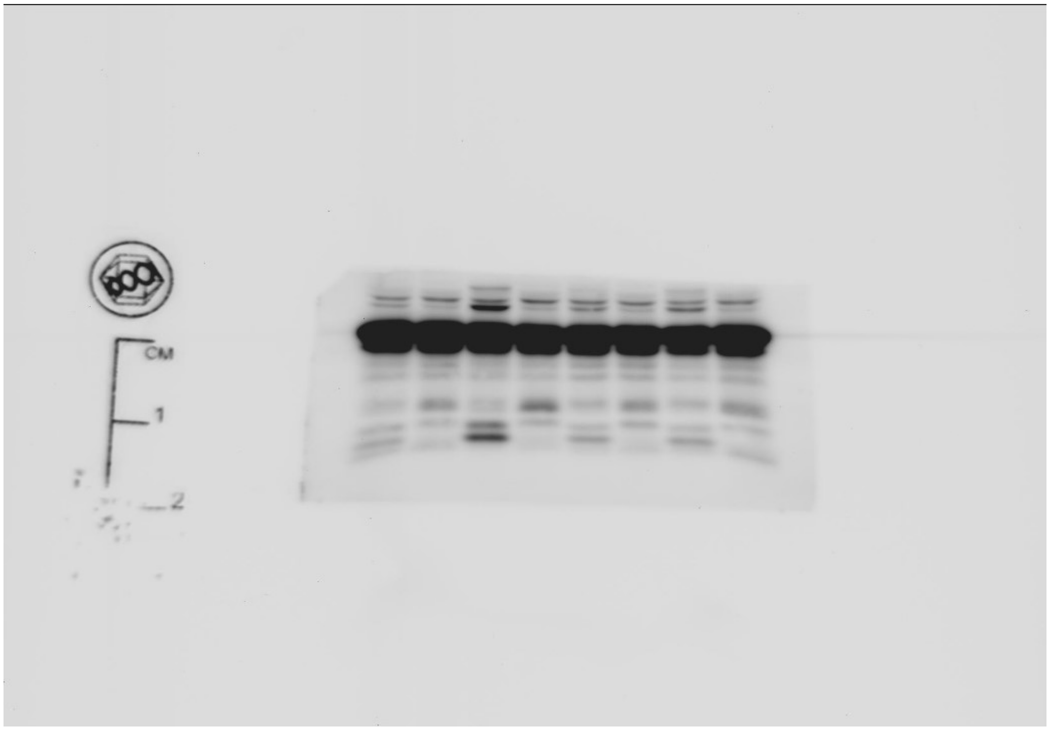

Figure S26. KMH-2/caspase-3.

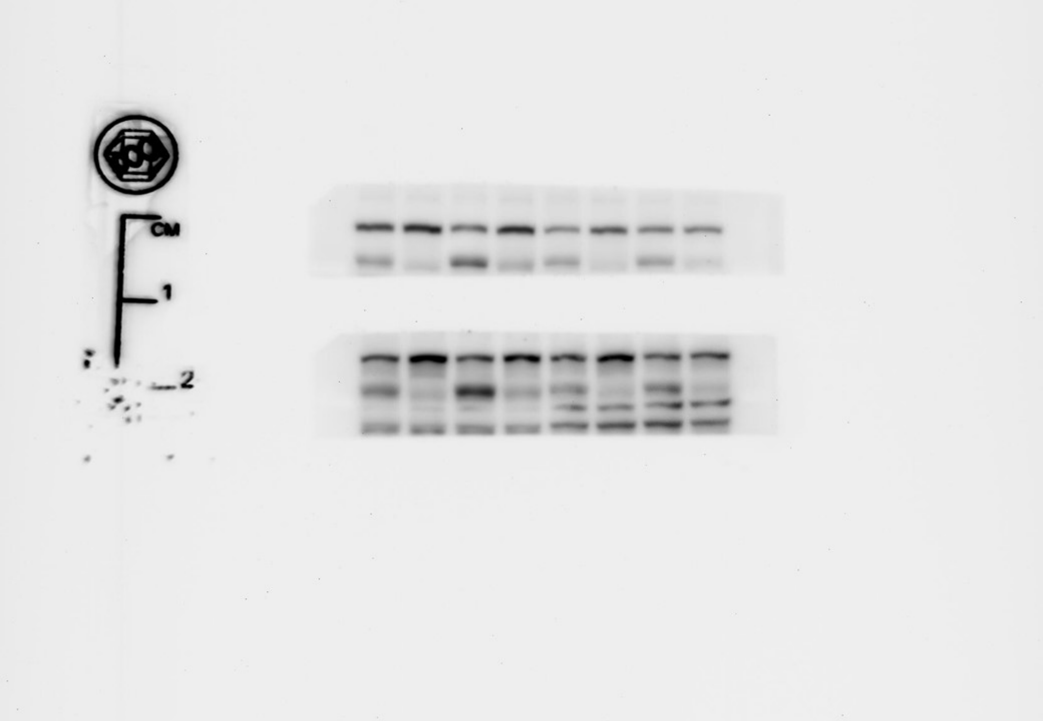

Figure S27. KMH-2/PARP-1.

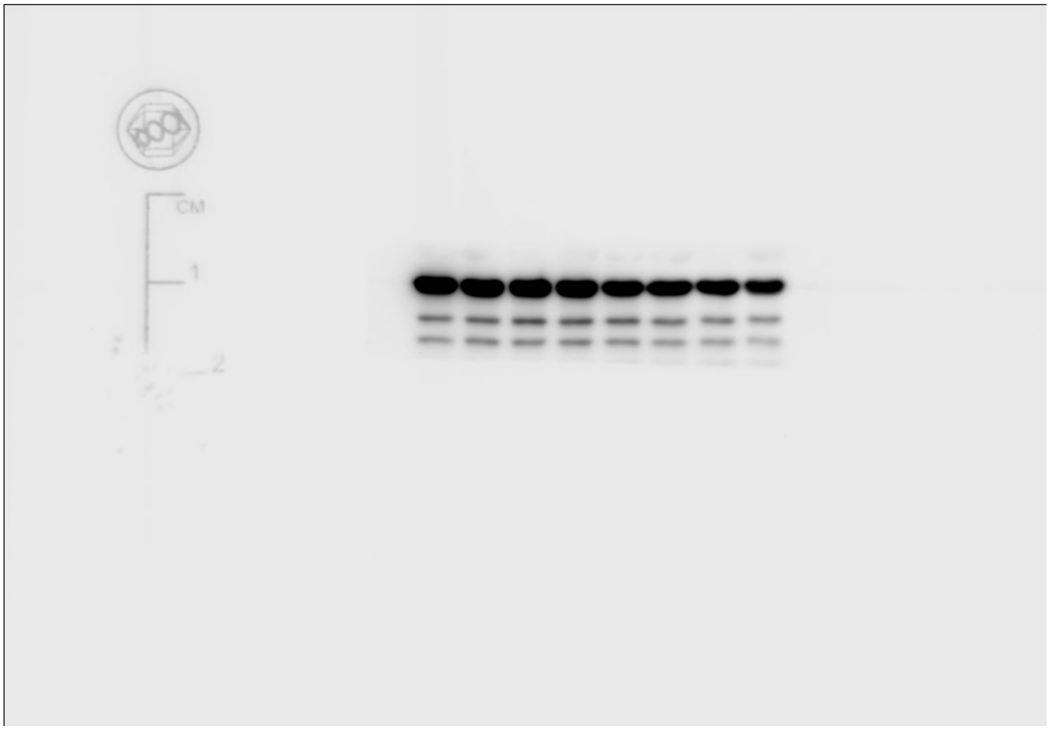

Figure S28. KMH-2/GAPDH.

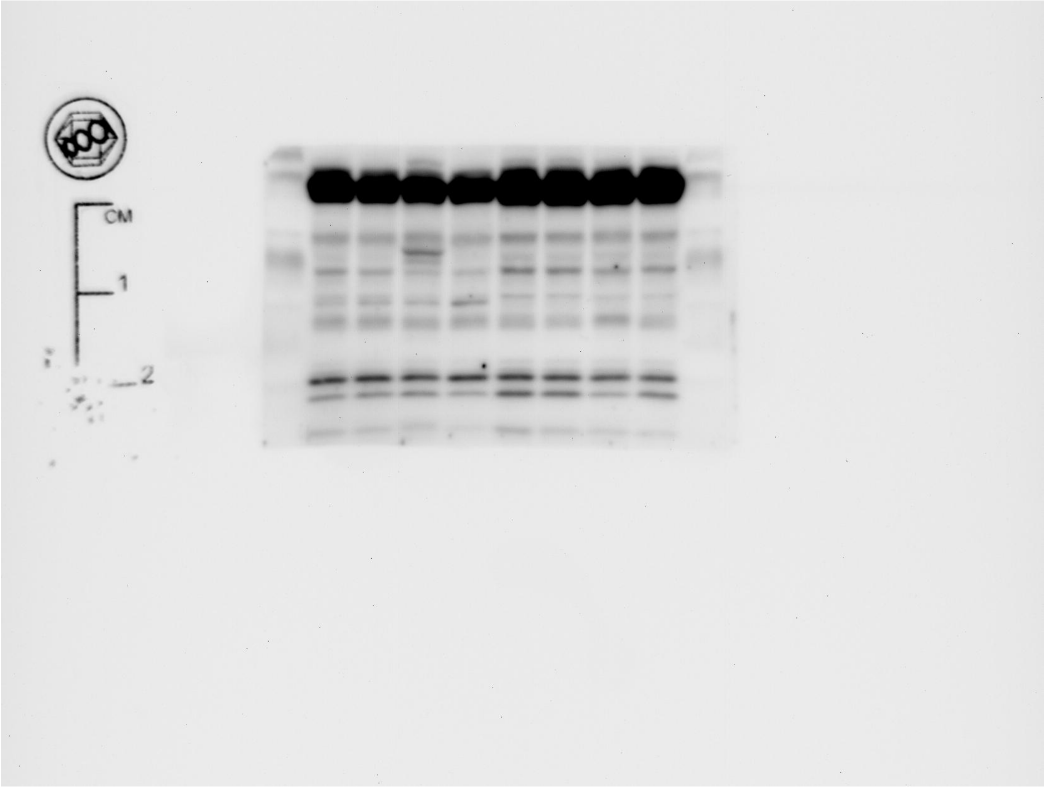

Figure S29. IHH-4/caspase-8.

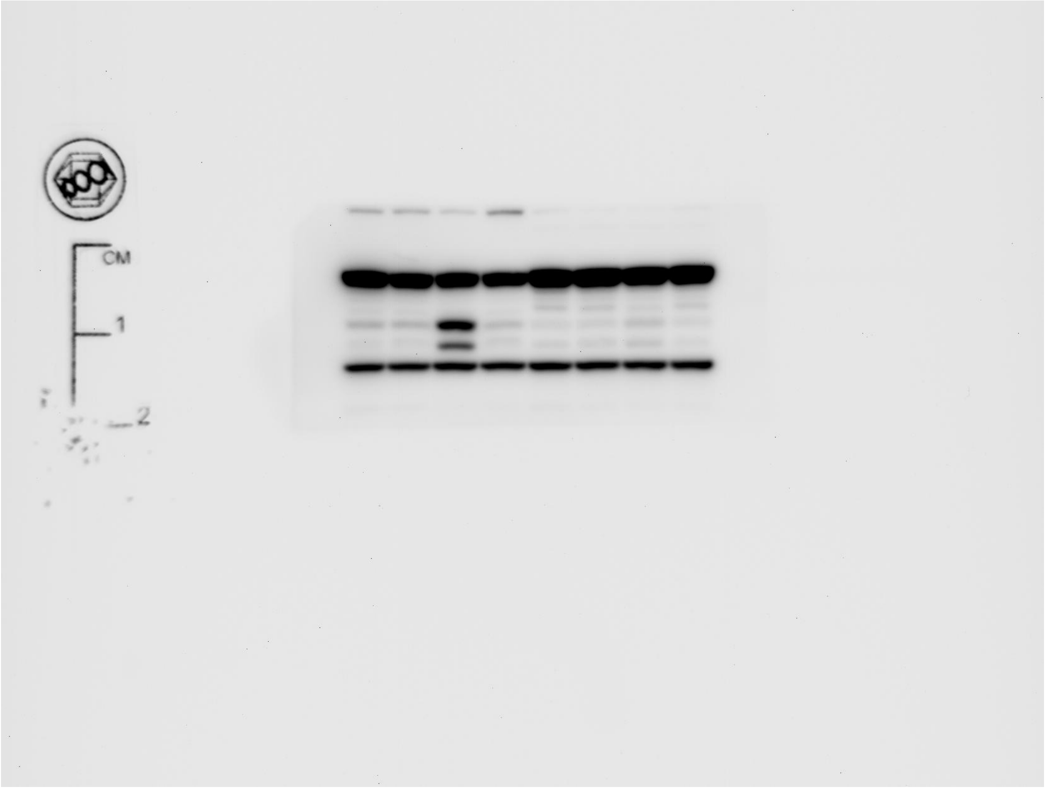

Figure S30. IHH-4/caspase-9.

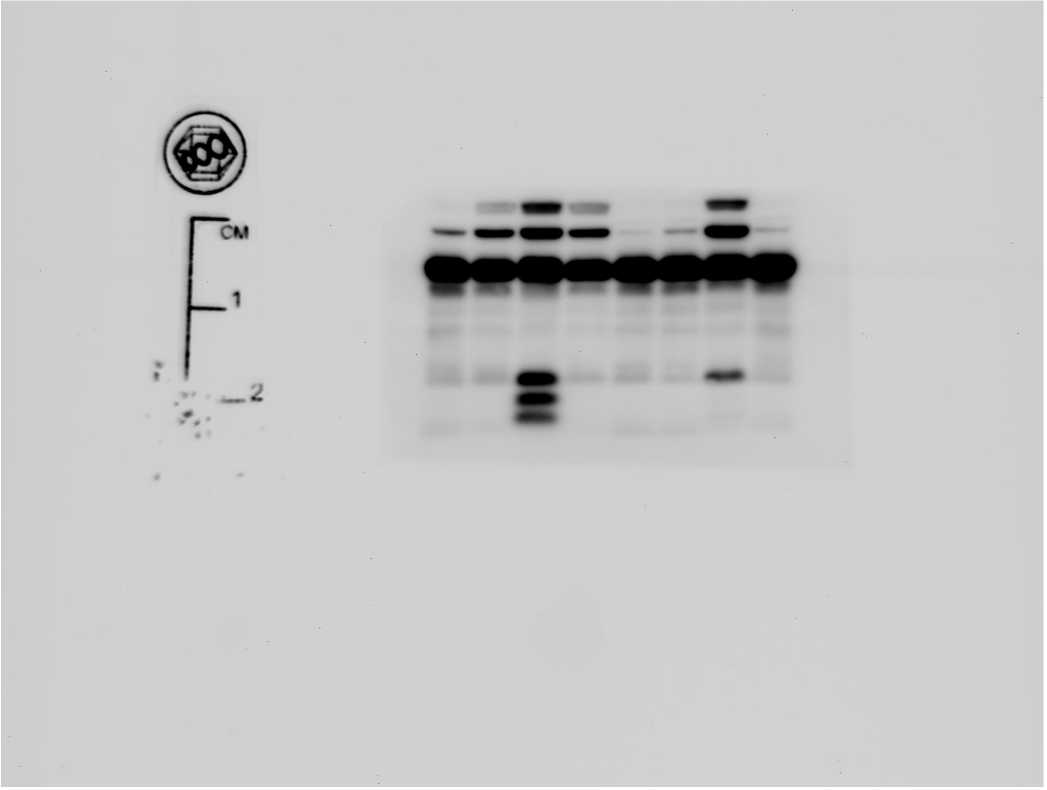

Figure S31. IHH-4/caspase-3.

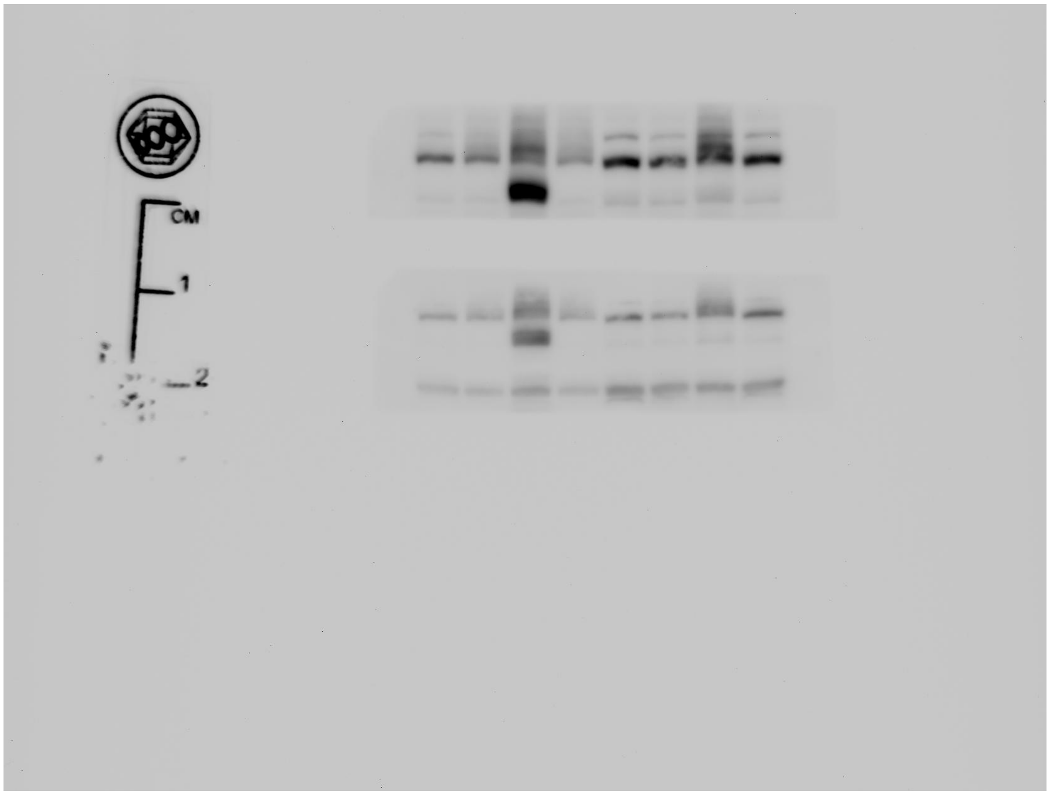

Figure S32. IHH-4/PARP-1.

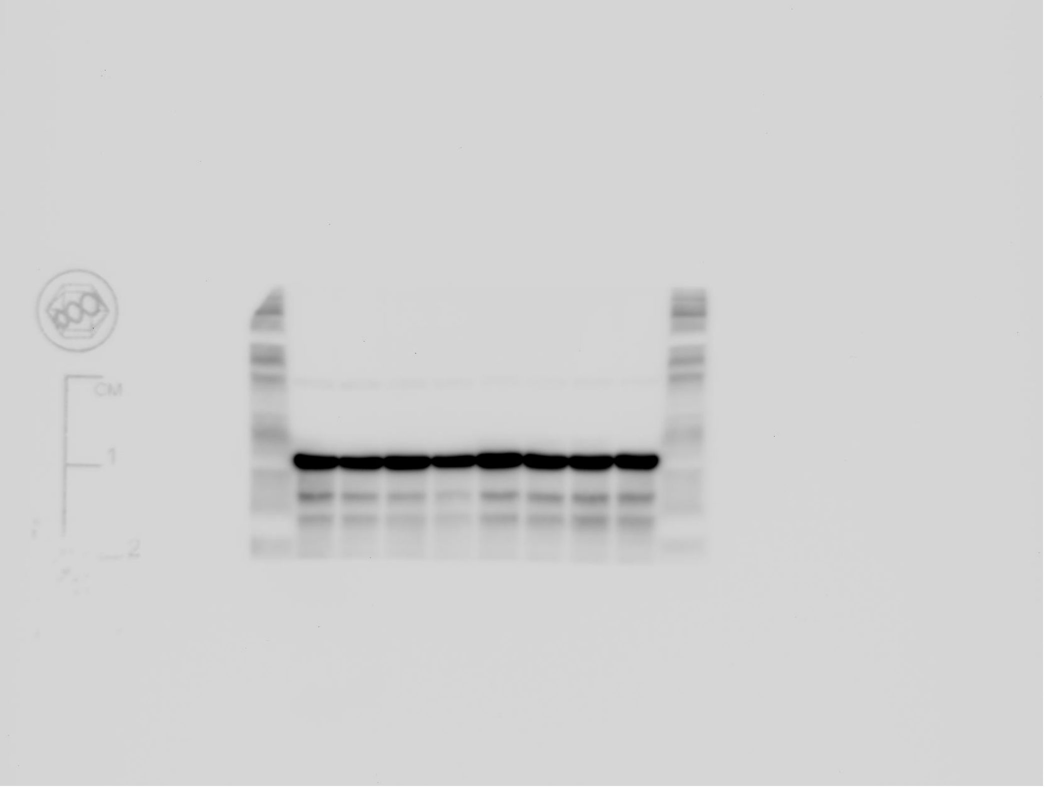

Figure S33. IHH-4/GAPDH.

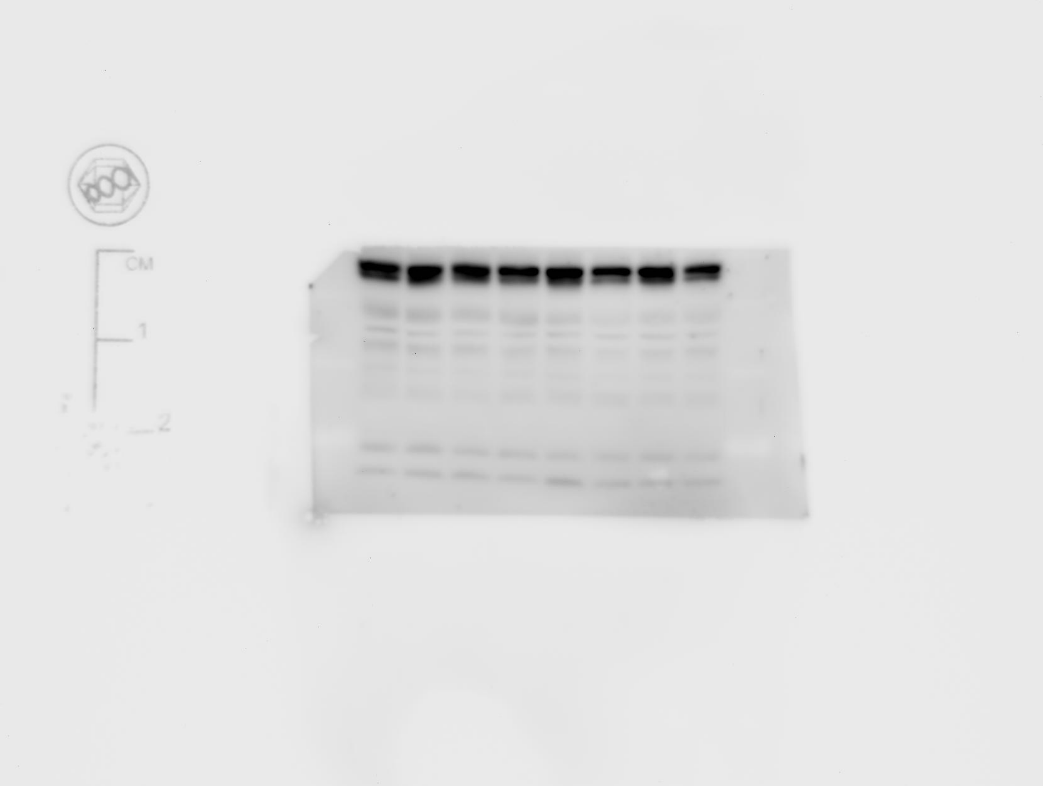

Figure S34. 8505c/caspase-8.

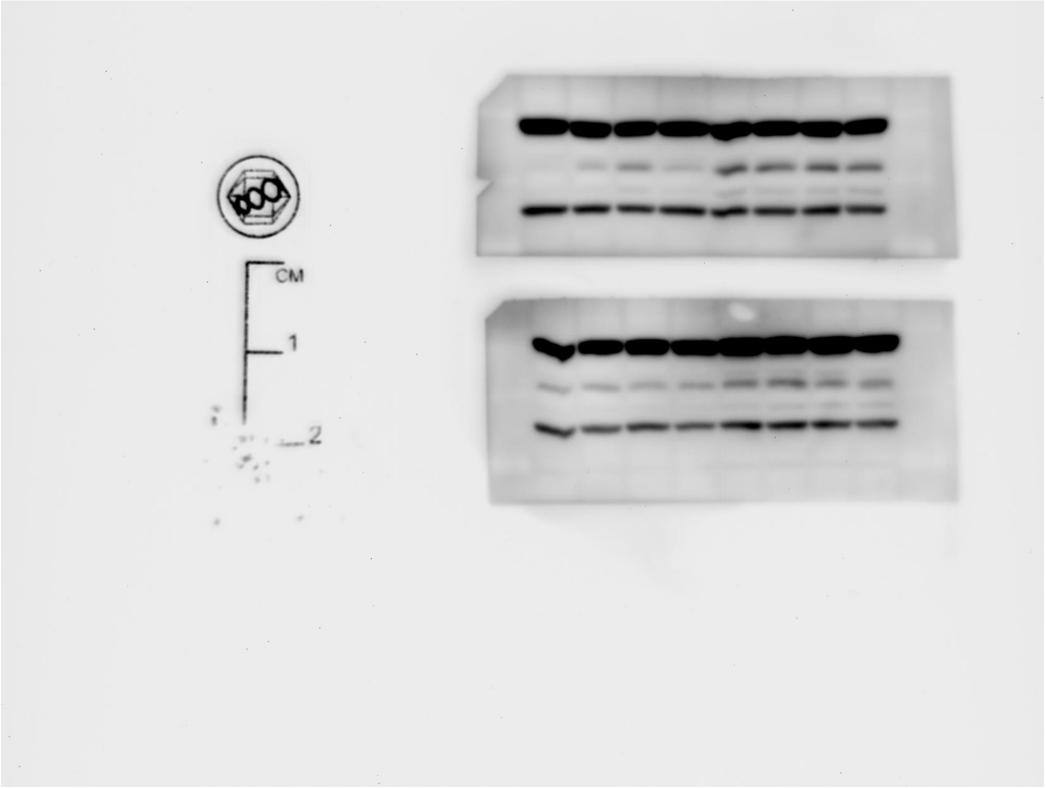

Figure S35. 8505c /caspase-9.

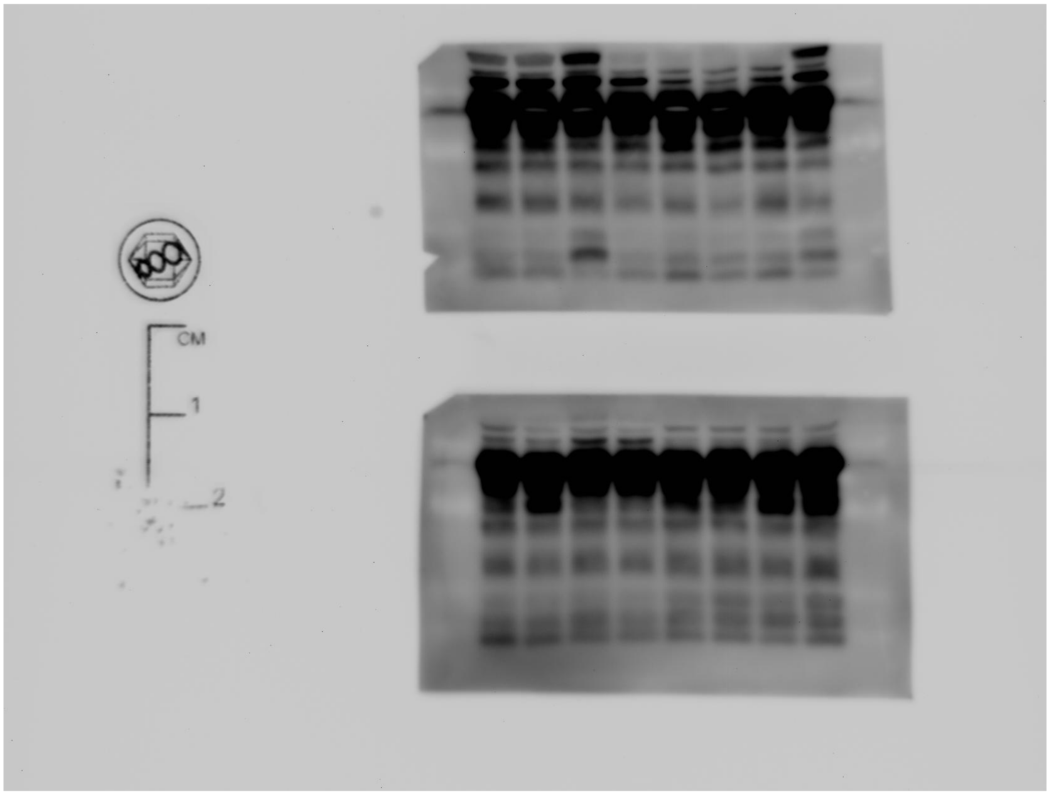

Figure S36. 8505c /caspase-3.

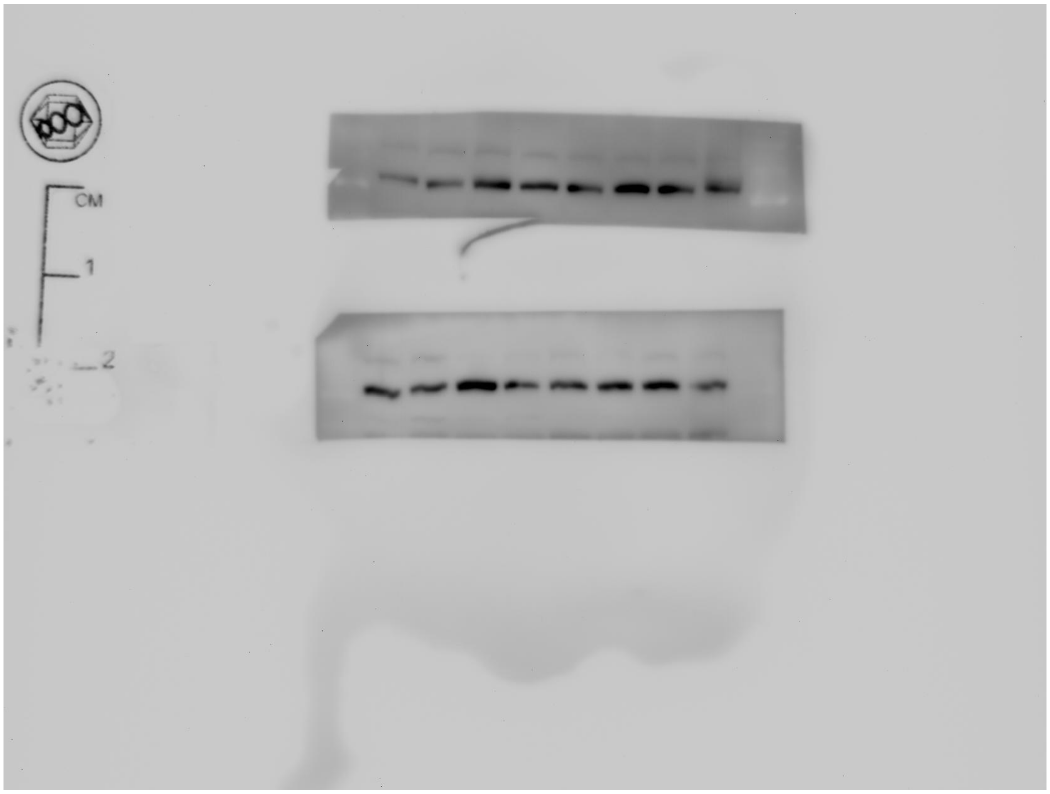

Figure S37. 8505c /PARP-1.

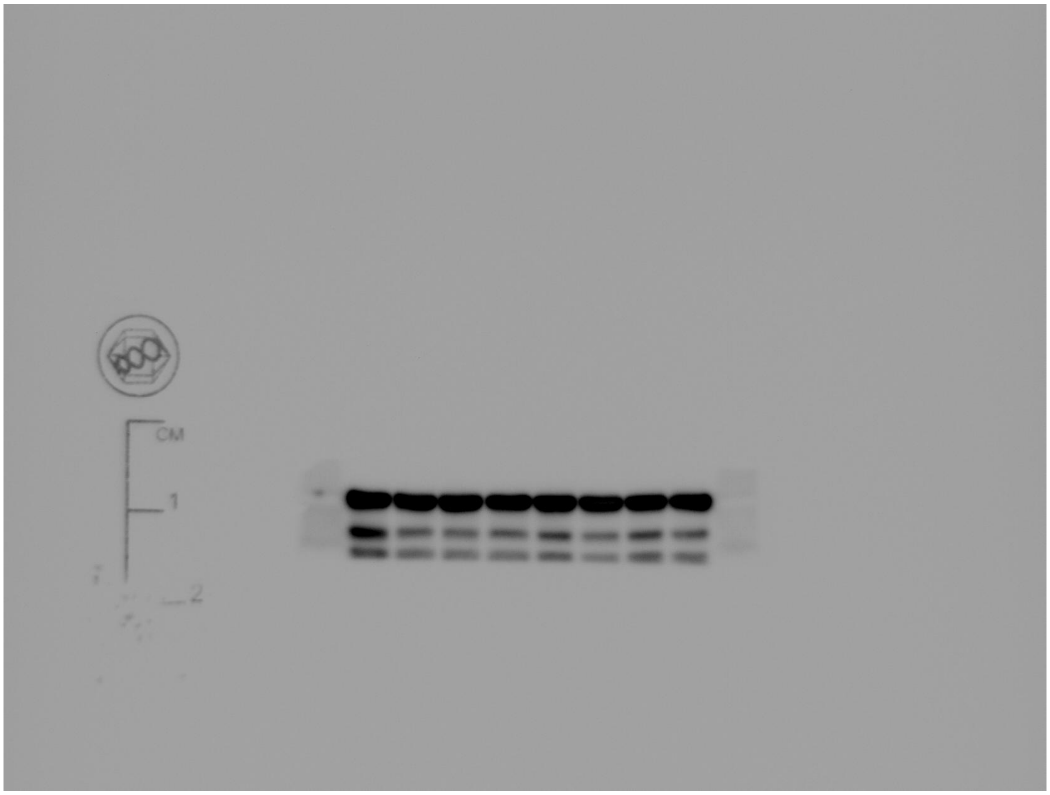

Figure S38. 8505c /GAPDH.

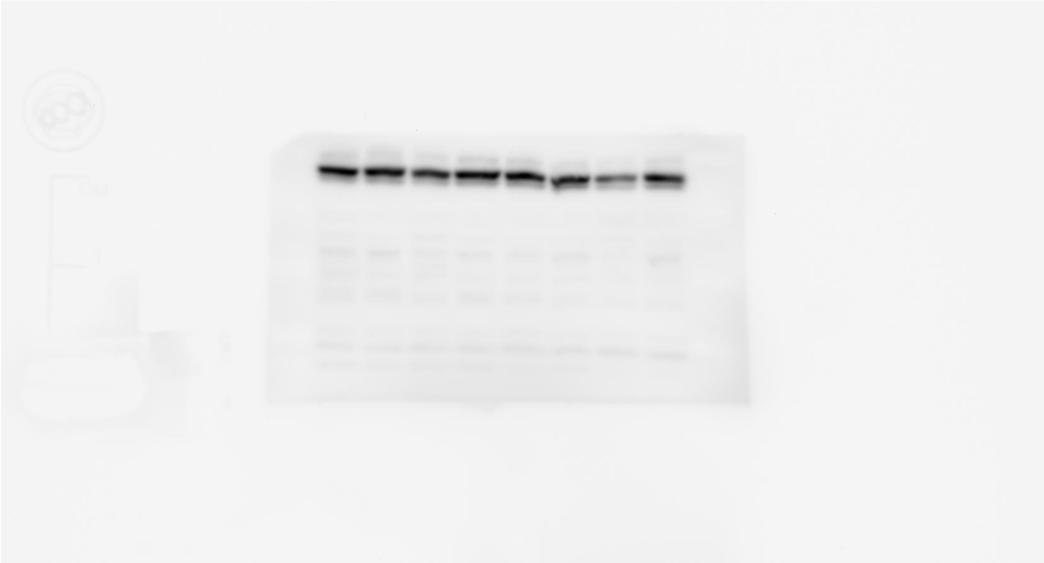

Figure S39. KMH-2/caspase-8.

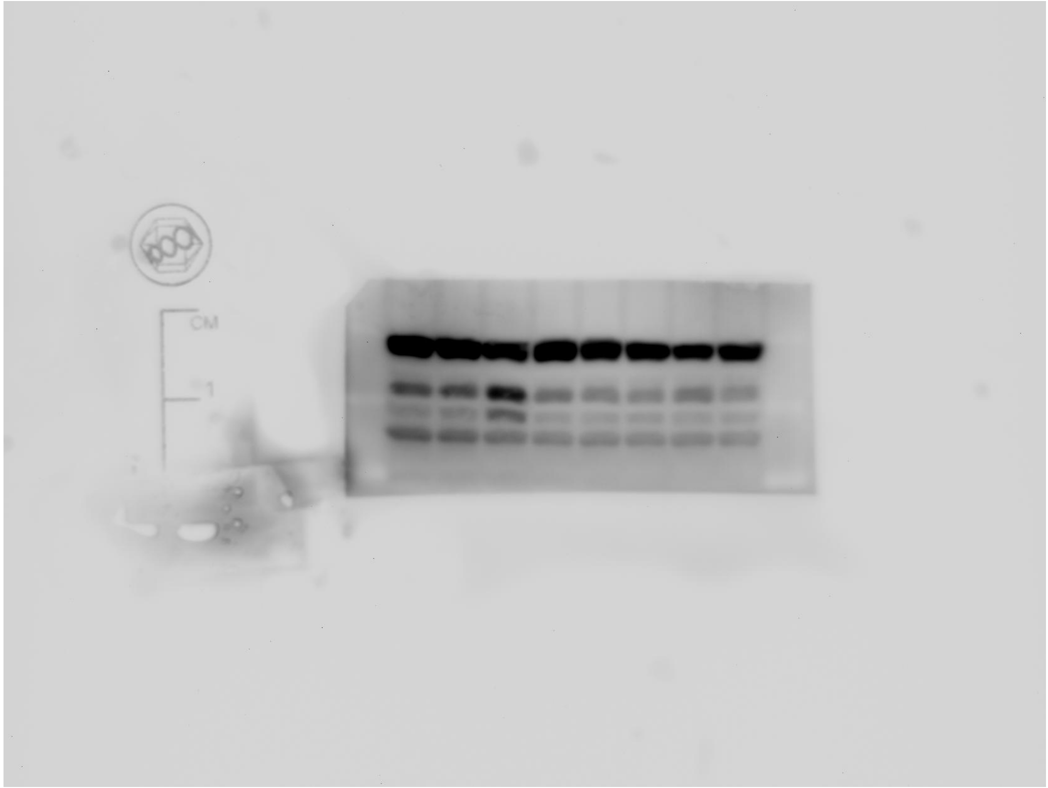

Figure S40. KMH-2/caspase-9.

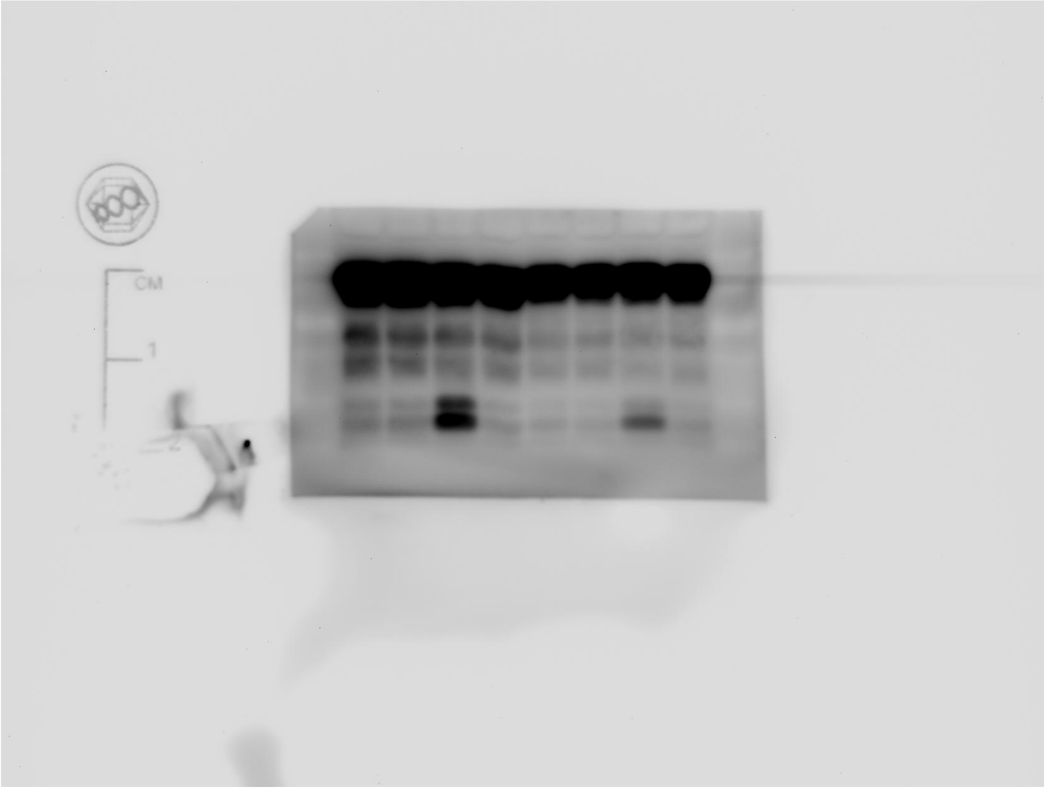

Figure S41. KMH-2/caspase-3.

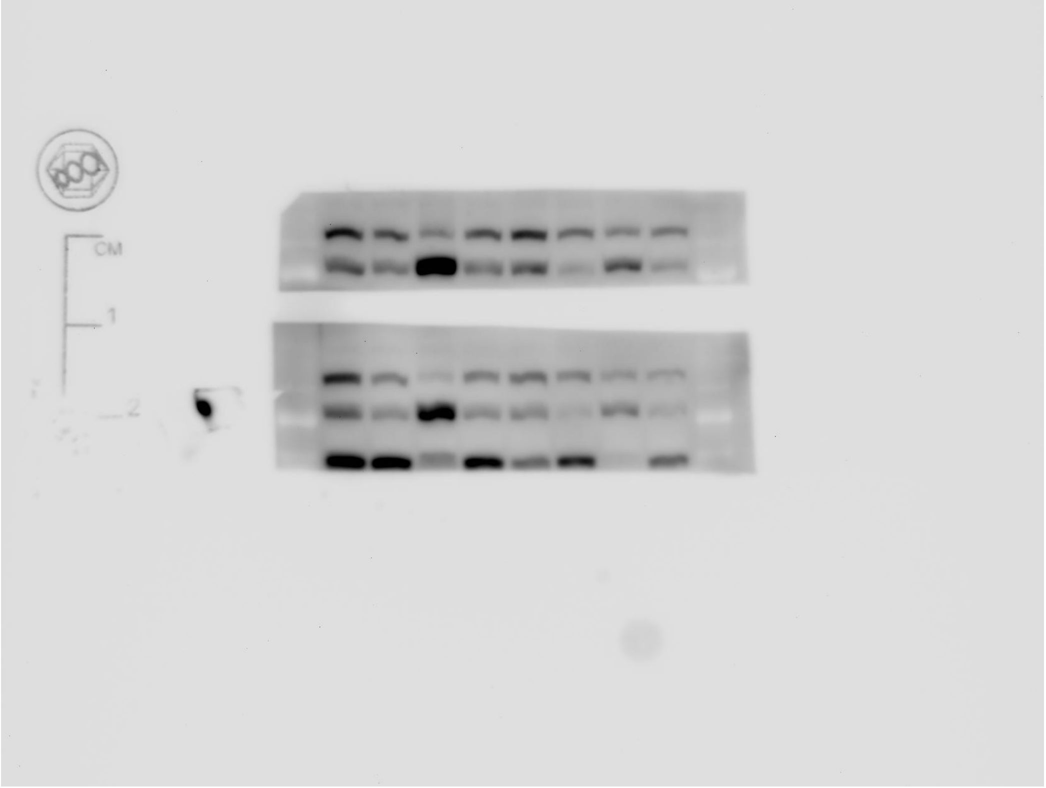

Figure S42. KMH-2/PARP-1.

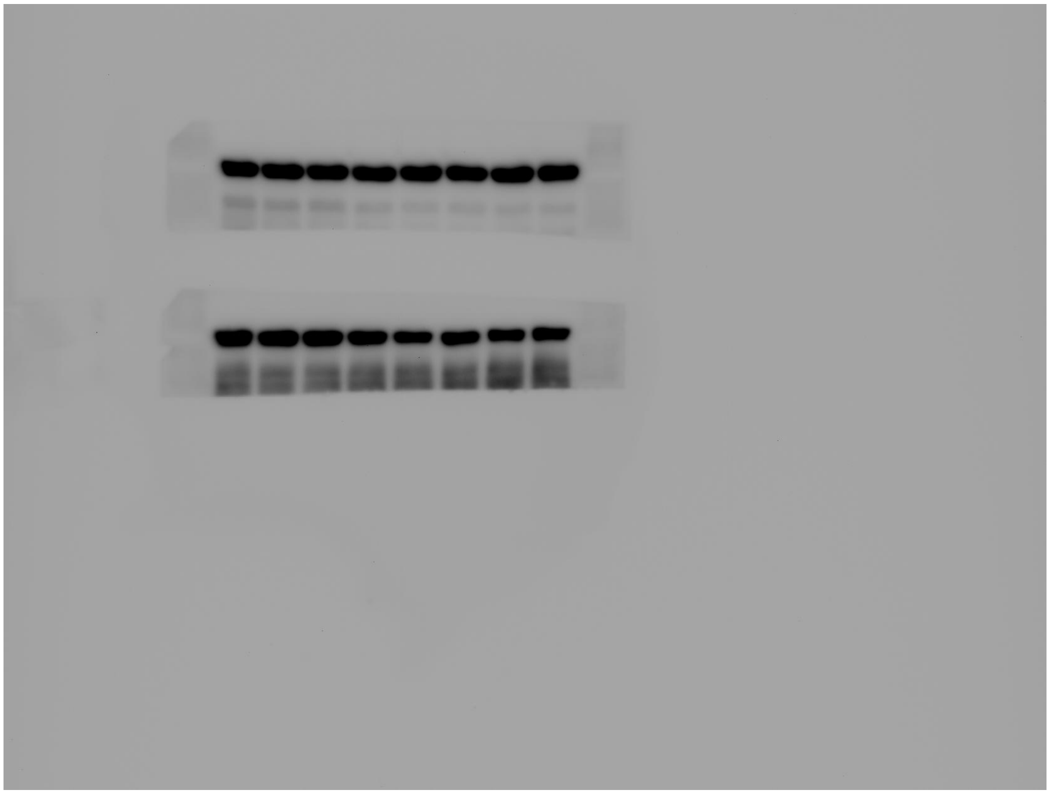

Figure S43. KMH-2/GAPDH.

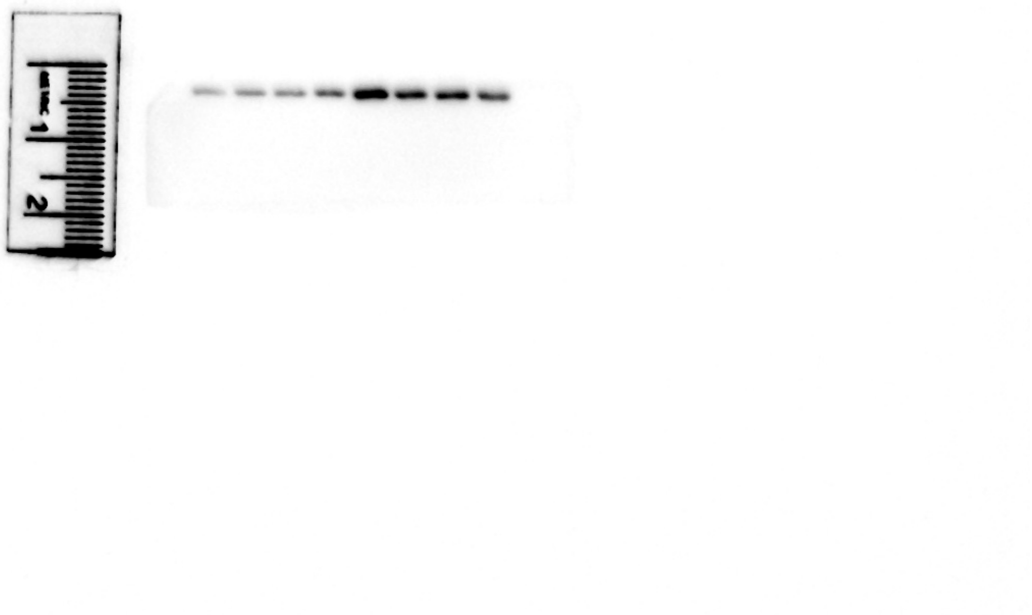

Figure S44. p-p38.

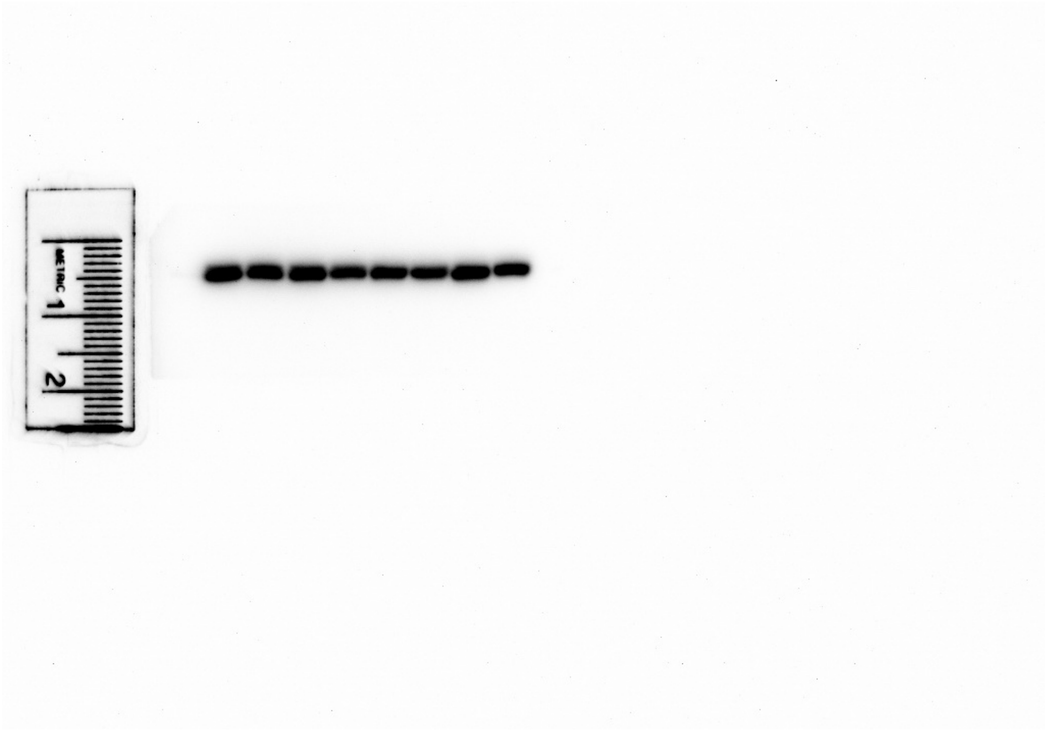

Figure S45. p38.

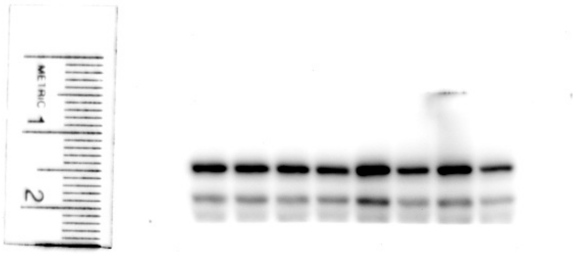

Figure S46. p-JNK.

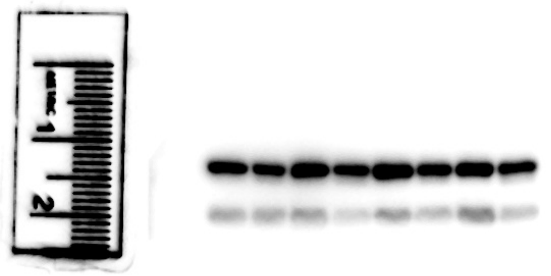

Figure S47. A. JNK.

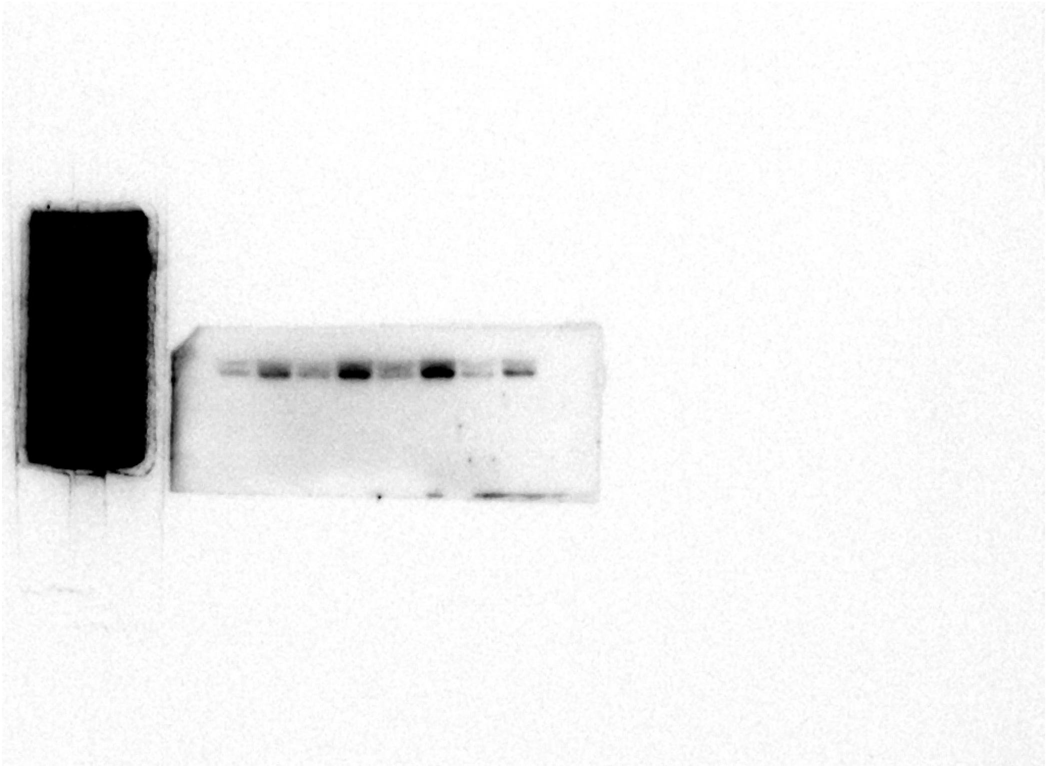

Figure S48. p-Erk.

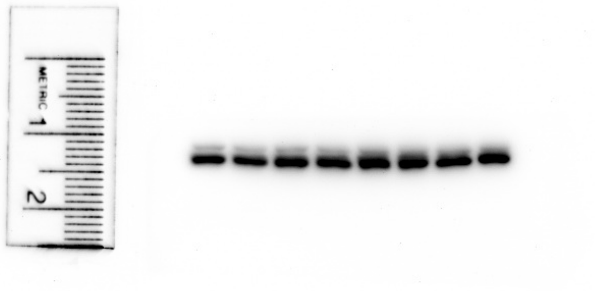

Figure S49. A. Erk.

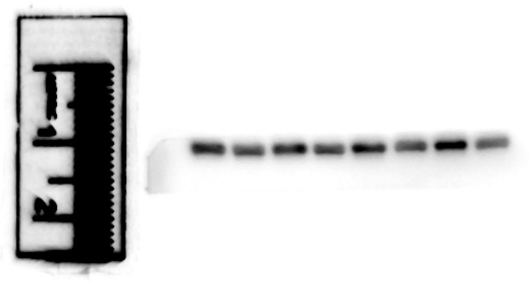

Figure S50. A. p-Akt.

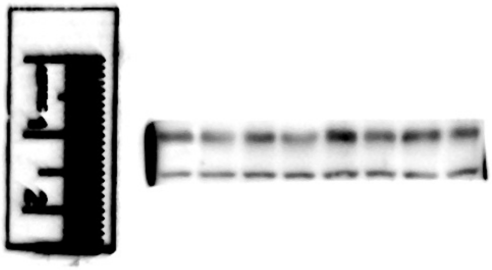

Figure S51. Akt.

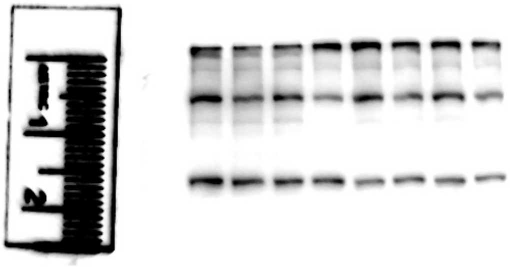

Figure S52. p-mTOR.

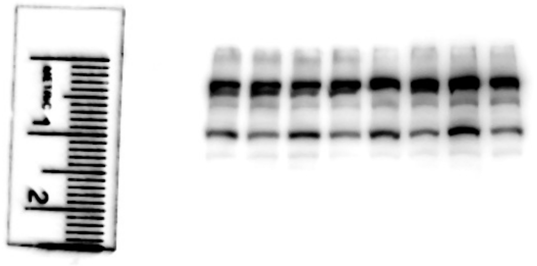

Figure S53. mTOR.

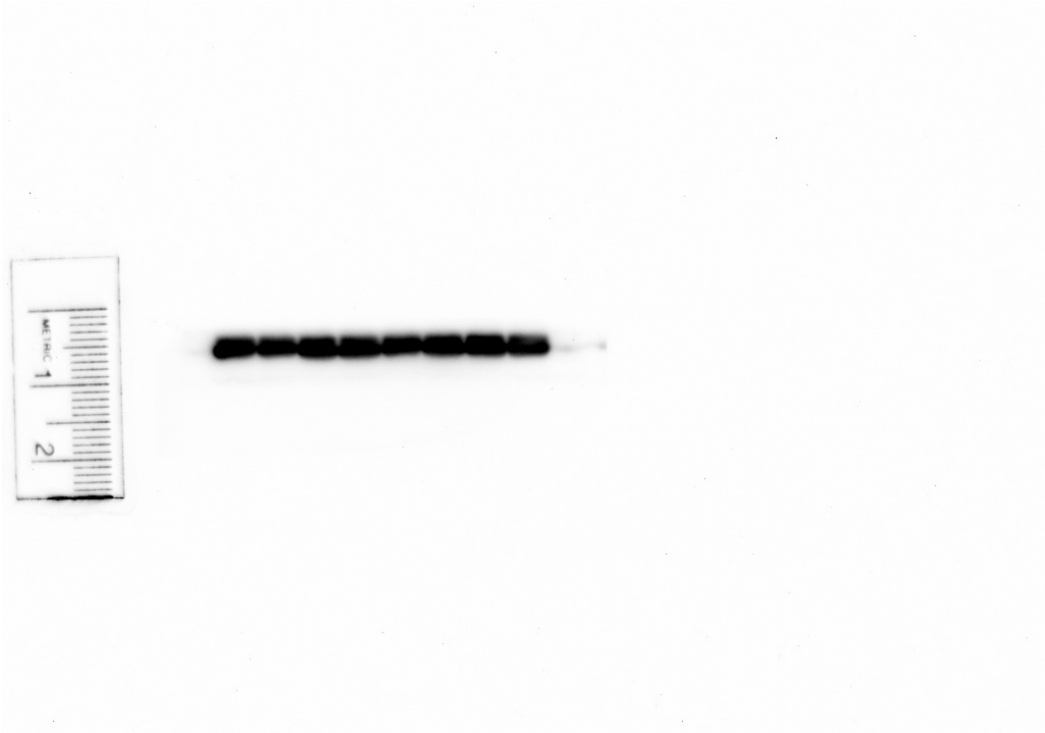

Figure S54. GAPDH.

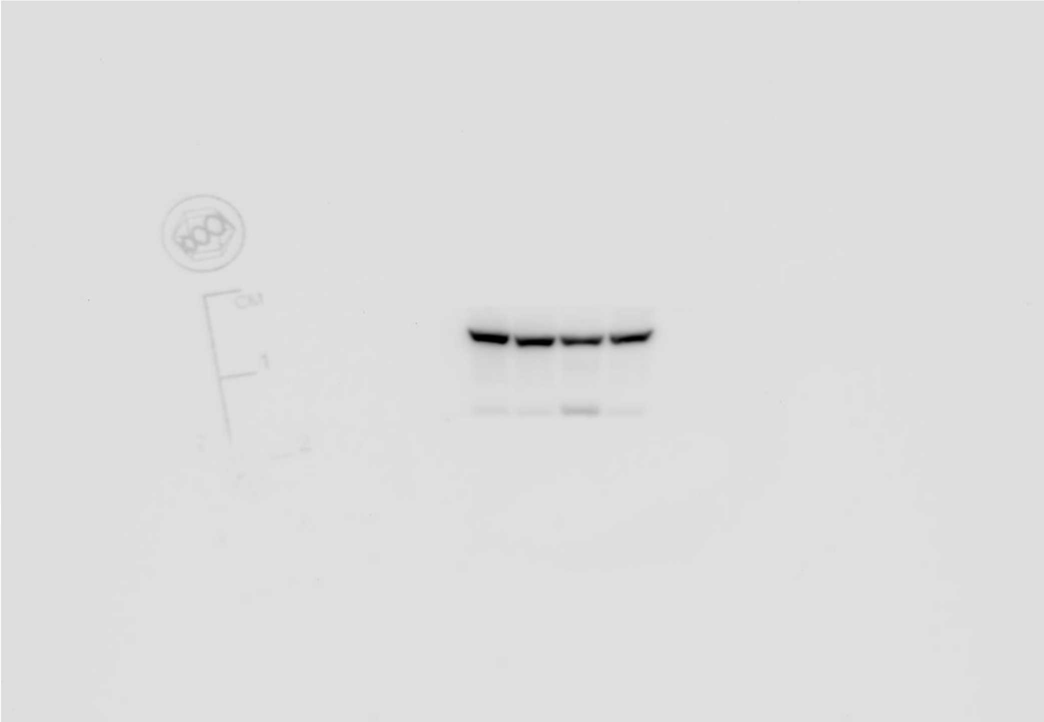

Figure S55. p-p38.

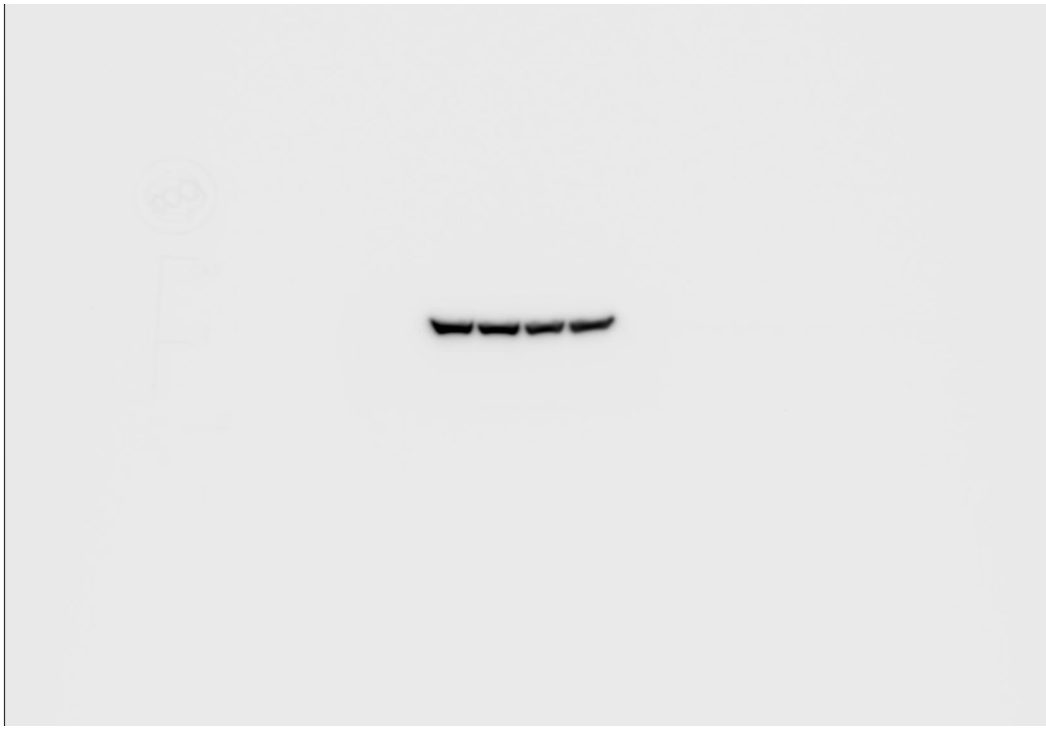

Figure S56. p38.

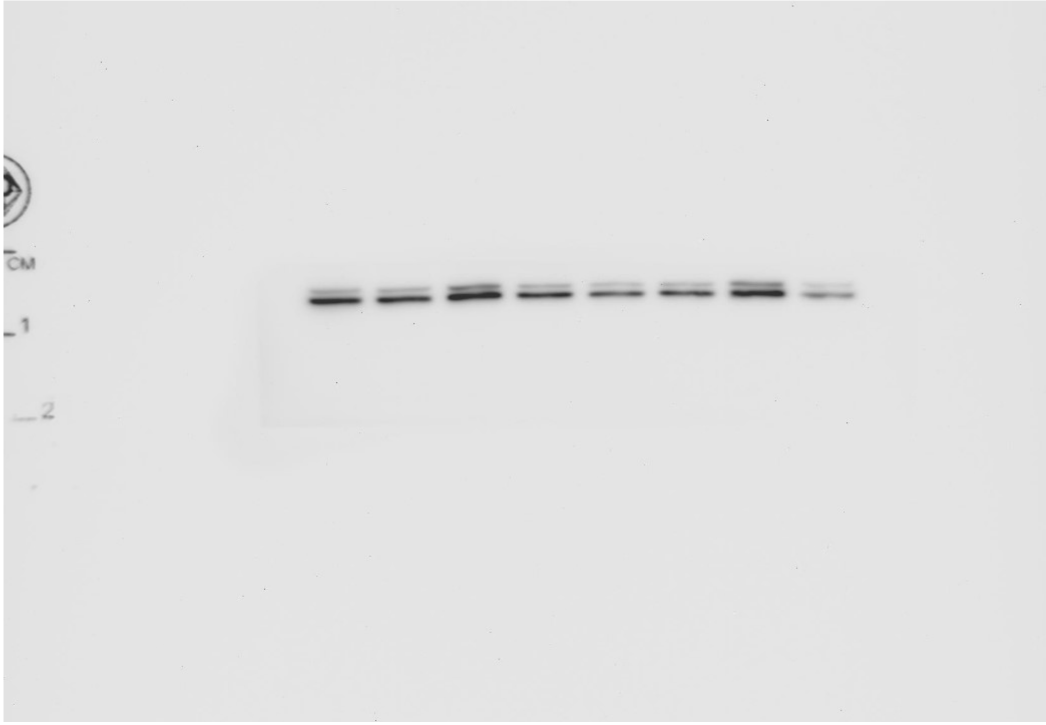

Figure S57. p-Erk.

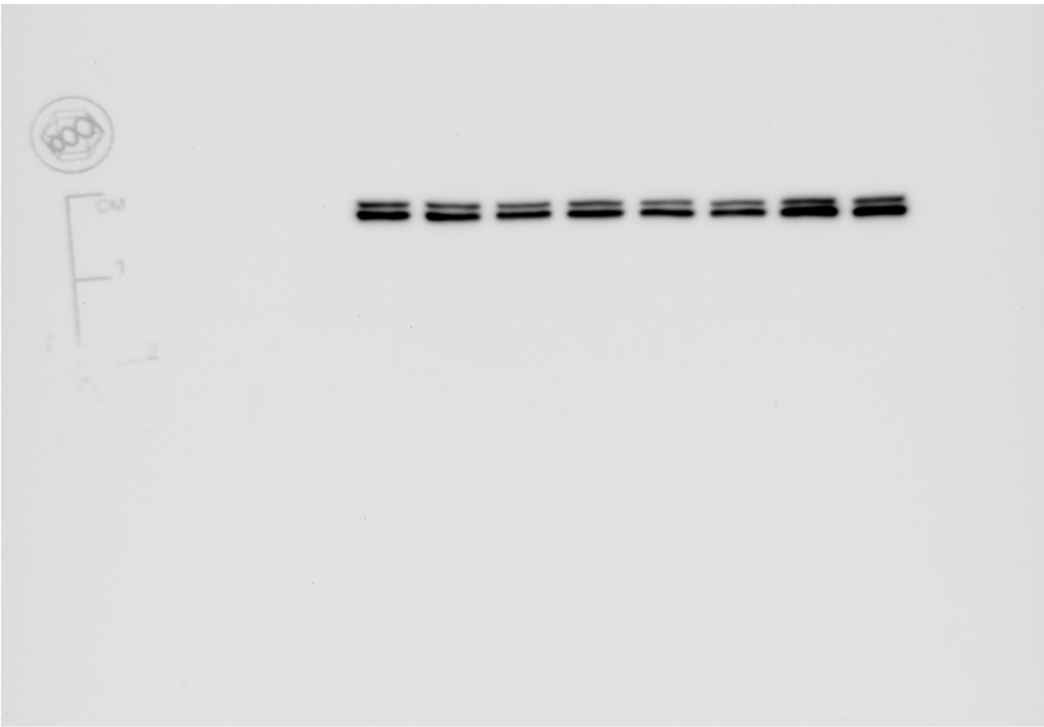

Figure S58. Erk.

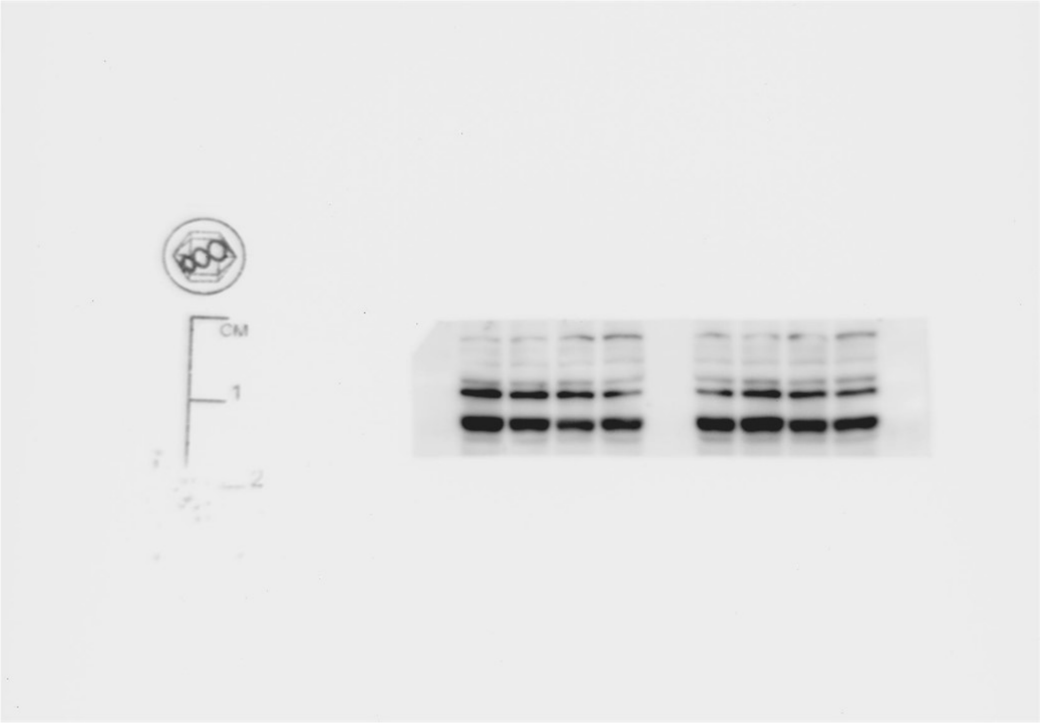

Figure S59. p-Akt.

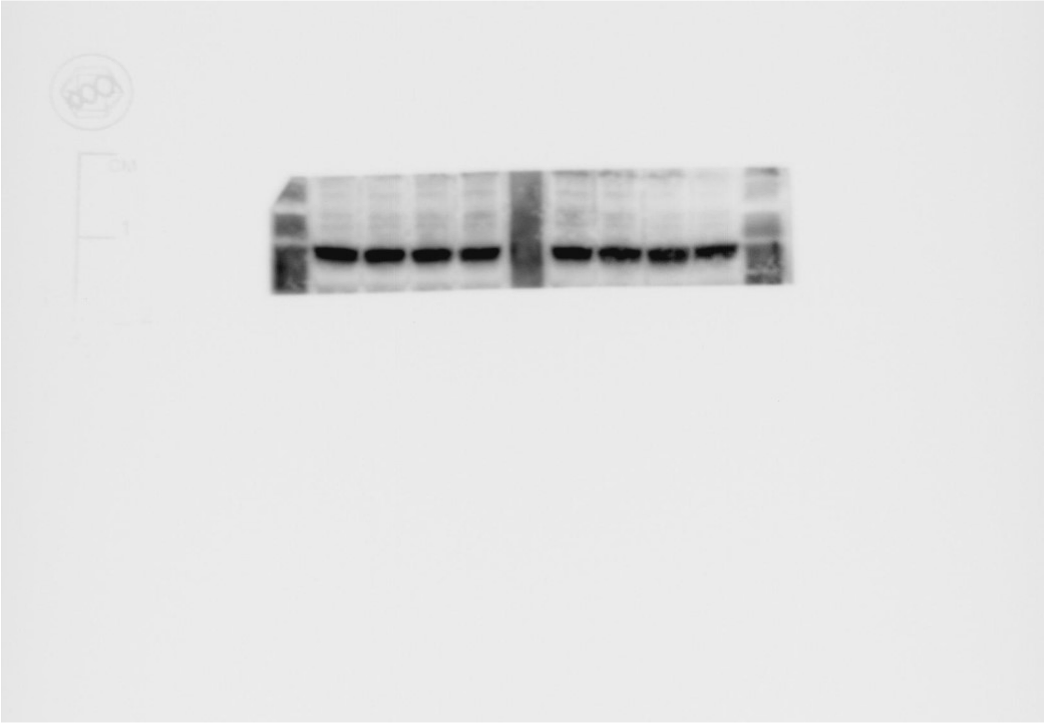

Figure S60. Akt.

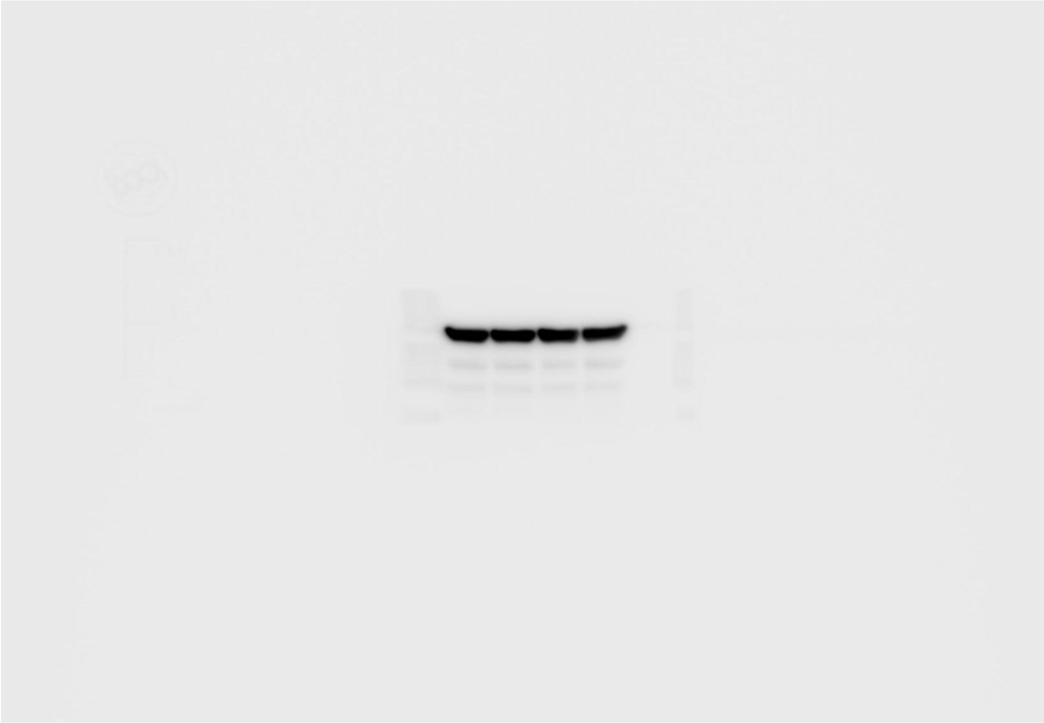

Figure S61. GAPDH.

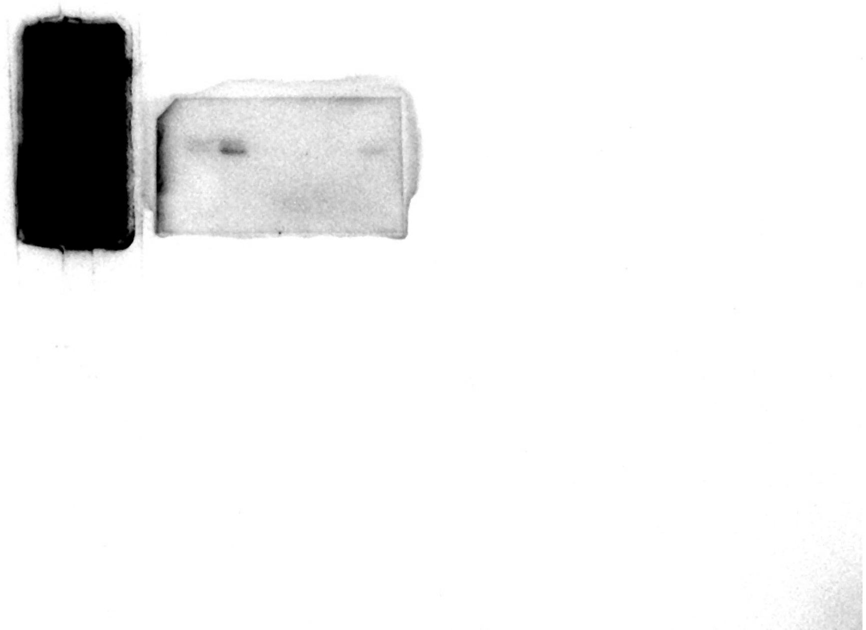

Figure S62. p-Erk.

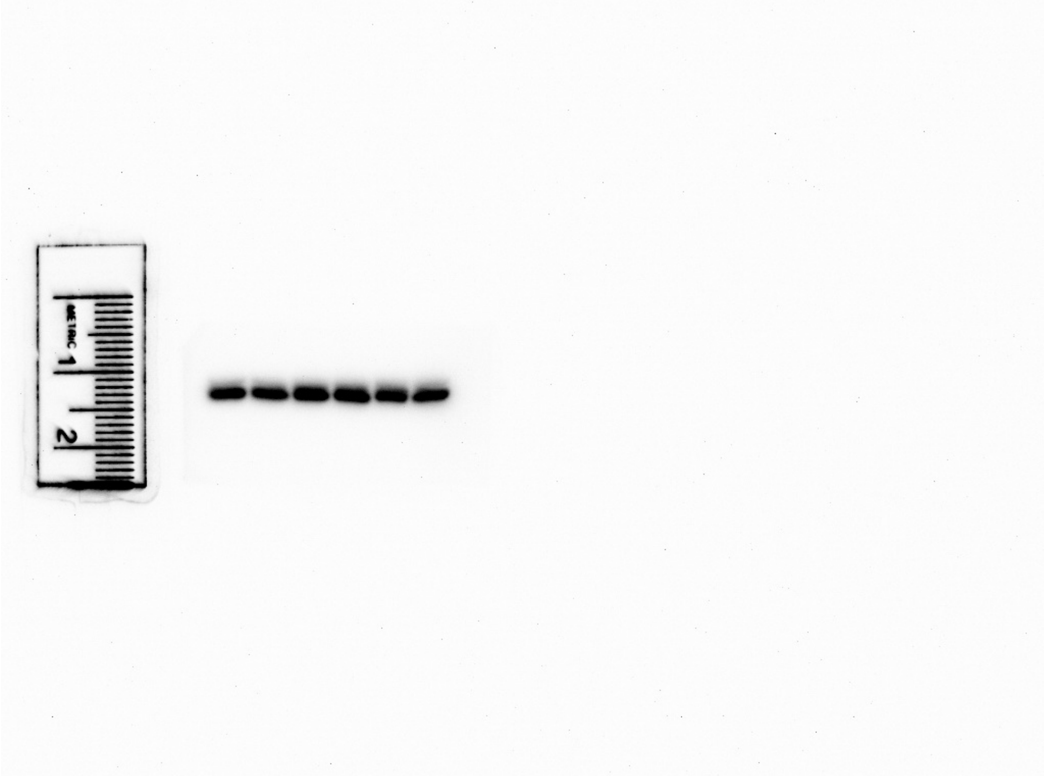

Figure S63. Erk.

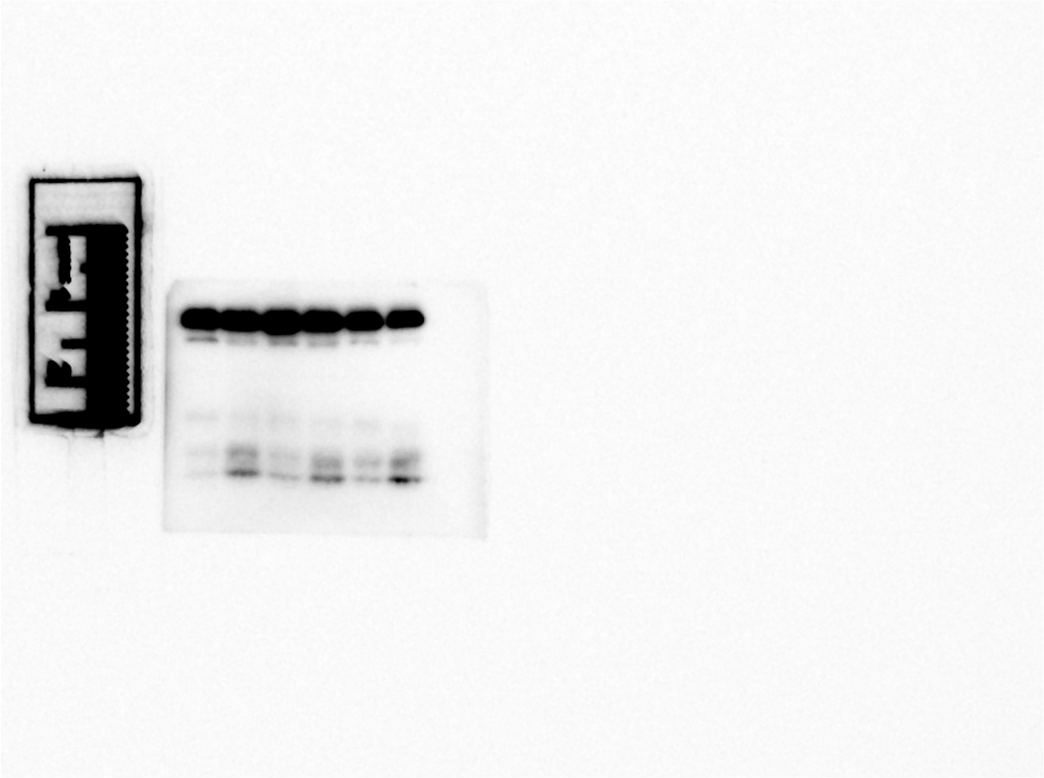

Figure S64. caspase-3.

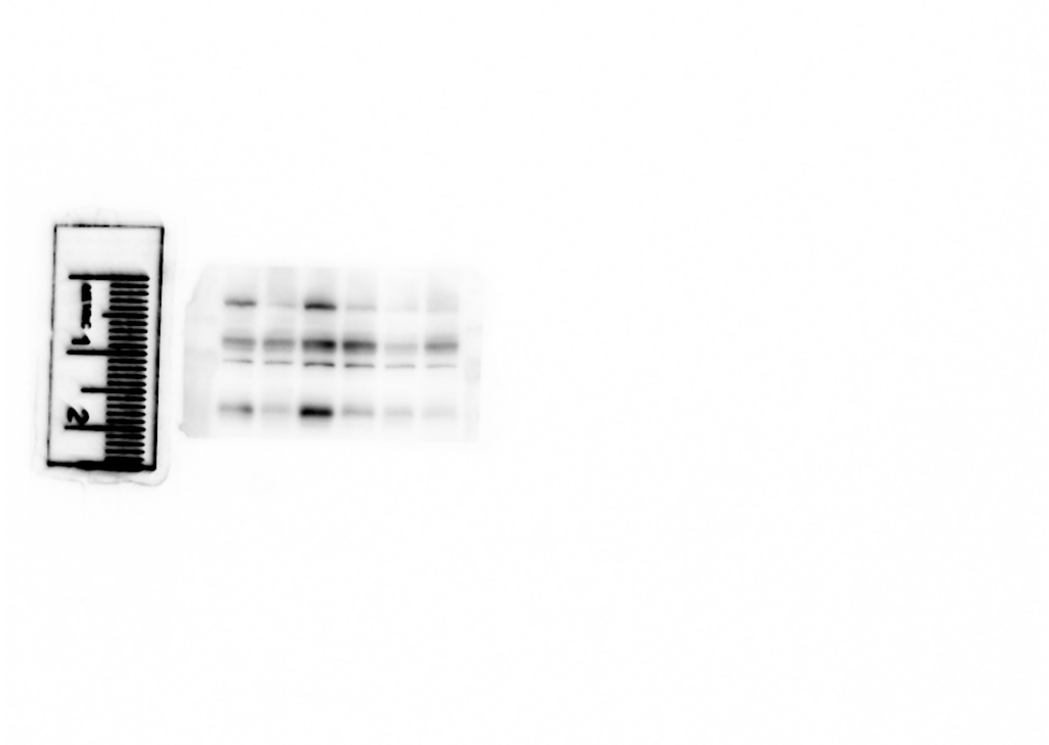

Figure S65. PARP.

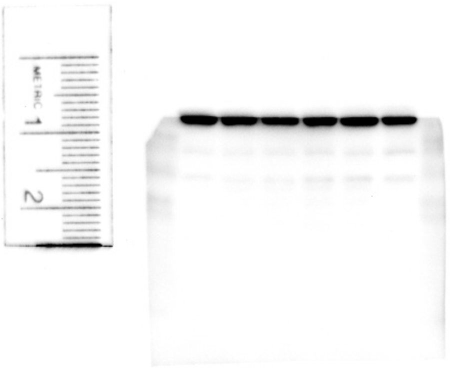

Figure S66. GAPDH.

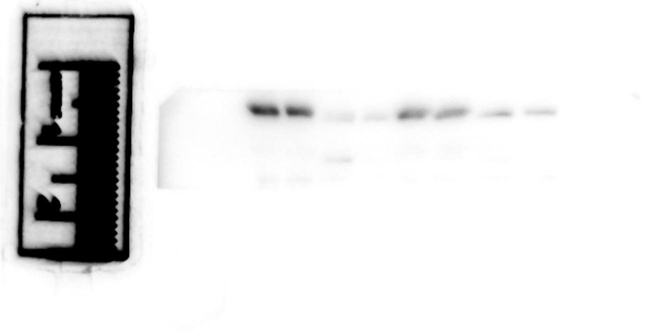

Figure S67. p-Akt.

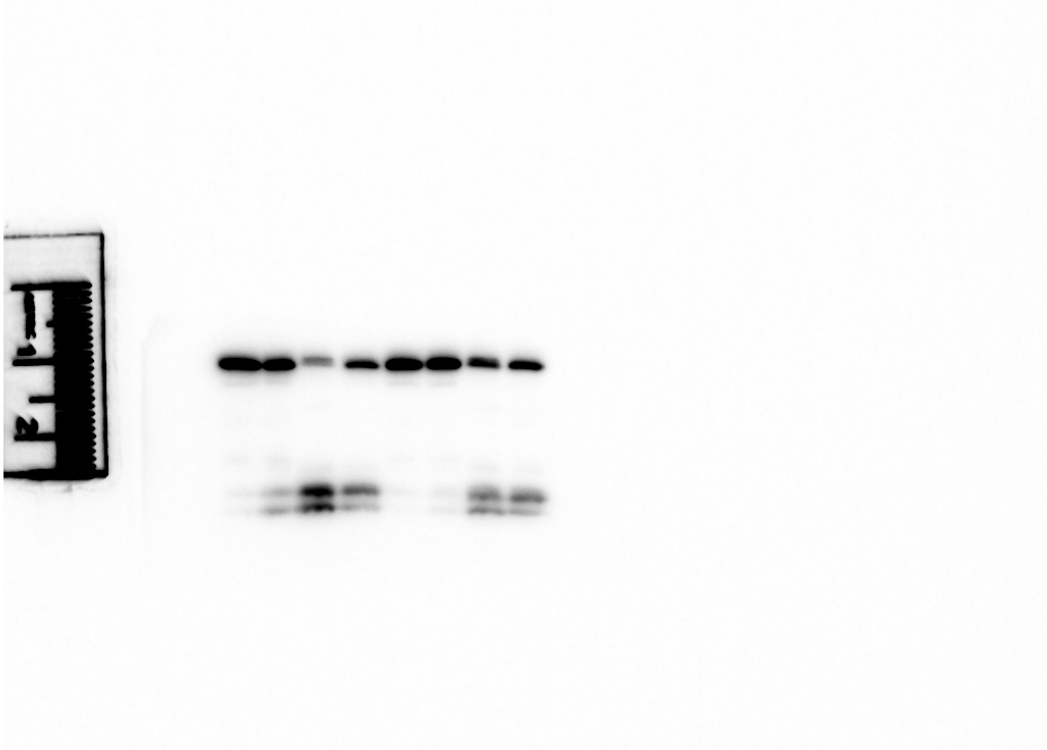

Figure S68. caspase-3.

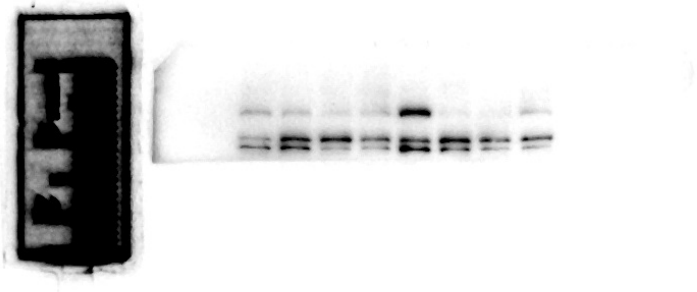

Figure S69. PARP.

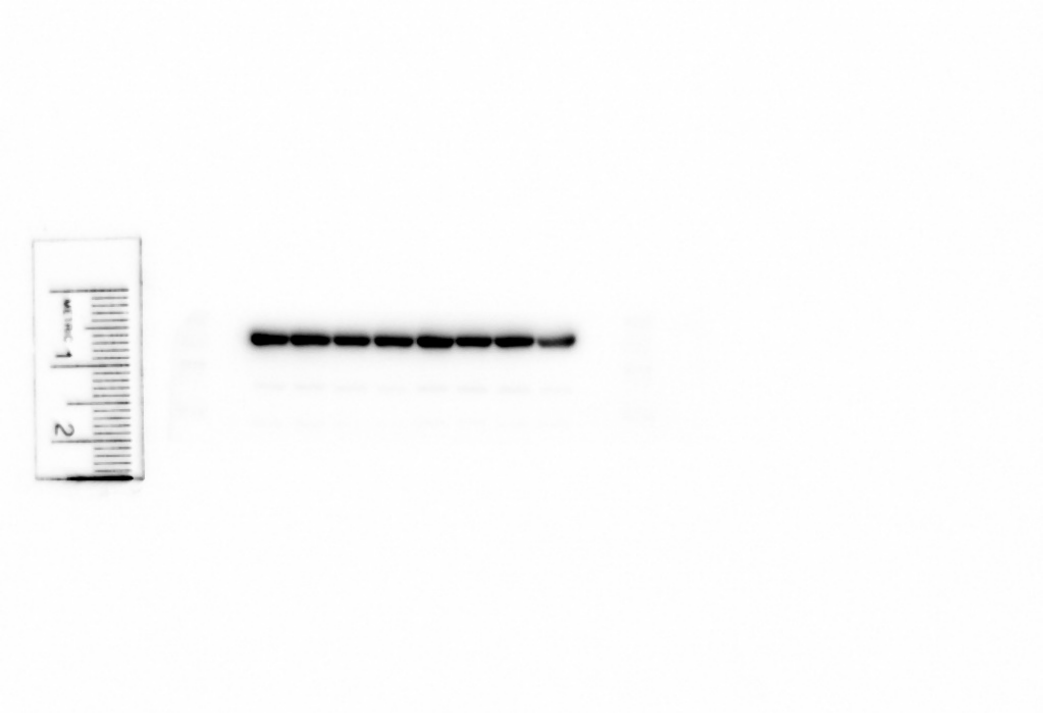

Figure S70. GAPDH.

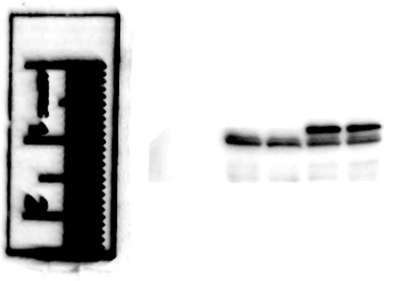

Figure S71. p-Akt.

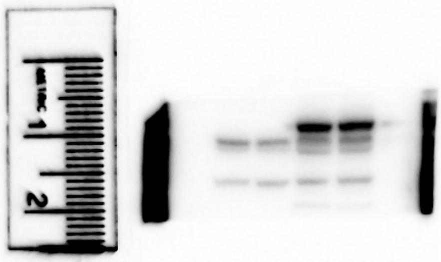

Figure S72. Akt.

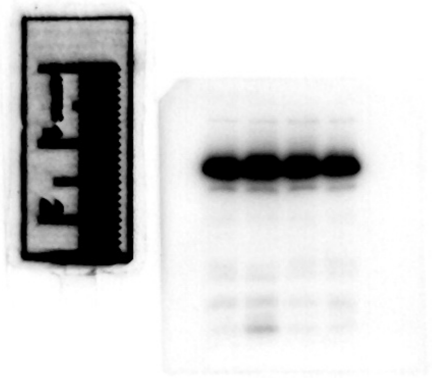

Figure S73. caspase-3.

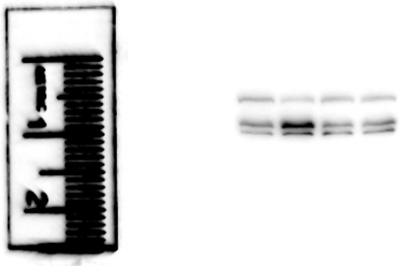

Figure S74. PARP.

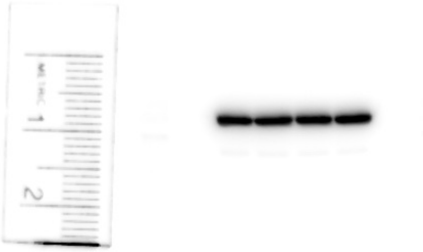

Figure S75. GAPDH.
